# Design and Optimization of a Gold and Silver Nanoparticle-Based SERS Biosensing Platform

**DOI:** 10.3390/s25041165

**Published:** 2025-02-14

**Authors:** Soumyadeep Saha, Manoj Sachdev, Sushanta K. Mitra

**Affiliations:** 1 Micro and Nanoscale Transport Laboratory, Department of Mechanical & Mechatronics Engineering, Waterloo Institute for Nanotechnology, University of Waterloo, Waterloo, ON N2L 3G1, Canada; 2 Department of Electrical & Computer Engineering, Waterloo Institute for Nanotechnology, University of Waterloo, Waterloo, ON N2L 3G1, Canada; msachdev@uwaterloo.ca

**Keywords:** nanoparticles, surface enhanced raman scattering (SERS), biosensor

## Abstract

This study investigates the design and optimization of a nanoparticle-based surface-enhanced Raman scattering (SERS) biosensing platform using COMSOL Multiphysics simulations. The primary goal is to enhance the sensitivity and specificity of SERS biosensors, which are crucial for the precise detection and quantification of biomolecules. The simulation study explores the use of gold and silver nanoparticles in various arrangements, including single, multiple, and periodic nanospheres. The effects of polarization and the phenomenon of local hotspot switching in trimer and tetramer nanosphere systems are analyzed. To validate the simulation results, a SERS biosensing platform is fabricated by self-assembling gold nanoparticles on a silicon substrate, with methylene blue used as the Raman probe molecule. The findings demonstrate the feasibility of optimizing SERS biochips through simulation, which can be extended to various nanostructures. This work contributes to the advancement of highly sensitive and specific SERS biosensors for diagnostic and analytical applications.

## 1. Introduction

Raman scattering is a powerful spectroscopic technique that leverages the inelastic scattering of photons by molecules to provide detailed information about their vibrational modes. This phenomenon has numerous applications in chemical and biological analysis due to its ability to generate unique spectral fingerprints for different molecular species [1]. The specificity of Raman spectroscopy arises from the distinct vibrational energy levels associated with various chemical bonds, making it possible to identify and characterize functional groups within complex organic molecules. Furthermore, Raman spectroscopy is not merely a qualitative tool; it can also provide quantitative insights into the concentration and distribution of analytes within a sample by analyzing the intensity of the Raman peaks [2]. This capability is particularly advantageous in biosensing applications, where precise detection and quantification of biomolecules are essential. By harnessing the detailed spectral information obtained from Raman scattering, one can develop highly sensitive and specific biosensors for a wide range of diagnostic and analytical purposes.

Surface-enhanced Raman scattering (SERS) leverages the amplification of Raman signals by incorporating metallic nanostructures designed to enhance the local electromagnetic fields via localized surface plasmons. This enhancement facilitates the detection of weak Raman signals, significantly boosting the sensitivity and specificity of biosensing applications. Extensive research has been conducted on SERS biosensing platforms with various noble metal nanostructures, including gold and silver. These studies have explored a range of nanoparticle geometries, from simple spherical nanoparticles [3,4,5] to more complex shapes such as nanorods [6,7], nanobowties [8,9,10], and nanoflowers [11]. Various SERS-based biosensors of innovative geometries have been reported in the literature, with a plethora of application regions. Zheng et al. (2023) [12] developed a tapered optical fiber decorated with gold nanoislands, which have shown a remarkable detection limit of as low as 10 μM for serotonin and dopamine, paving the way for in situ neurotransmitter probing. On the other hand, Lim et al. (2020) [13] fabricated mesoporic silver films using self-assembled polymeric micelles under controlled conditions and were able to get a detection limit of 1 nM with the Raman probe molecule Rhodamine 6G. Self-assembly processes are generally simpler in terms of fabrication; however, this often results in uncontrolled structural dimensions if performed with limited conditional changes.

While many such existing studies have demonstrated remarkable success in detecting trace amount of biomolecules, they face challenges like reproducibility and uniformity in nanostructure fabrication, which can lead to variability in the enhancement factor. Additionally, the use of complex nanostructures often requires sophisticated fabrication techniques that are not easily scalable for practical applications. Another significant drawback is the potential for photothermal heating effects, particularly with high-intensity laser excitation, which can cause the degradation of the analyte or the nanostructure itself.

The amplification or enhancement factors of a SERS biochip modified with metallic nanostructures depend on various parameters such as nanostructure size and shape, interstructure nanogaps, and incident wavelength [14,15,16,17]. All these factors influence the resonance of the localized surface plasmon, which provides the local electric field enhancement. Therefore, it is necessary to determine the correct design parameters of a SERS biochip beforehand in order to maximize its sensitivity. In this study, we discuss a simple model implementation of nanoparticle-based SERS biosensing platform in COMSOL Multiphysics [18] using finite element modeling (FEM) [19]. FEM has been widely accepted for SERS nanostructures simulations due to its ability to resolve complex structures where analytical solutions are difficult to obtain. Existing literature like Saini et al. (2022) [20], McMohan et al. (2009) [21], Micic et al. (2003) [22], and others have already implemented FEM for simulation studies of SERS nanoparticles, as well as other complex SERS geometries. In this study, comprehensive simulations were performed with both gold and silver material, taking into consideration different single, multiple, and periodic nanosphere systems. The polarization dependency of enhancement factor has been studied in order to understand the effect of orientation in regularly structured SERS platforms. An interesting phenomenon of local hotspot switching is observed in multiple nanosphere systems trimer and tetramer, which has not yet been reported in the literature. The implemented model could be very useful to optimize design parameters of a SERS biochip, and can be easily extended to various nanostructures, both simple and complex. In order to validate the simulation results, a simple SERS biosensing platform is fabricated by self-assembling gold nanoparticles on a silicon substrate. Methylene blue is used as the Raman probe molecule of choice for the validation of enhancement factor obtained from the periodic nanoparticle array simulation.

## 2. Materials and Methods

### 2.1. Materials

Sulphuric acid ($H_{2}SO_{4}$), hydrogen peroxide ($H_{2}O_{2}$), ACS grade acetone ($CH_{3}COCH_{3}$), and methylene blue powder were purchased from Fisher Chemicals, Massachusetts, USA. Ethanolamine ($HOCH_{2}CH_{2}NH_{2}$) was purchased from Alfa Aesar, Massachusetts, USA, and dimethyl sulfoxide (DMSO) ($CH_{3}SOCH_{3}$) was purchased from Sigma Aldrich, Massachusetts, USA. A suspension of gold nanoparticles (100 nm diameter) OD 1 stabilized in 0.1 mM PBS was purchased from Aldrich Chemistry. The phosphate buffer saline (PBS) at pH 7.4 was purchased from Gibco by Life technologies, California, USA. Silicon (100) wafers of 1 µm total thickness with a top oxide layer of 300 nm were purchased from Alpha Nanotech, Vancouver, Canada.

### 2.2. Fabrication

Initially, the silicon samples were cleaned in a 3:1 piranha solution of 98% concentrated sulphuric acid (30 mL) with 3% hydrogen peroxide (10 mL) for 15 min to remove any organic substances on the surface. The cleaned samples were then ultrasonicated with acetone for 20 min to remove any dust particles and impurities. The cleaned substrates were dried over a heating surface at 110 °C. Substrates were further processed through an air plasma treatment (320 W, 8 ccm, 150 mT) for 10 min for further cleaning and the activation of surface dangling bonds for amine functionalization in the next step.

Gold nanoparticles can be self-assembled over a Si surface via simple physisorption. However, the weakly physisorbed nanoparticles have a higher chance of getting washed out in future processing steps. Therefore, it is necessary to decorate the Si surface with a functional group that can form chemical bonds with Au. Ethanolamine is a good choice for the self-assembly of gold and silver nanoparticles. The −OH end of ethanolamine anchors with SiO_2_, while the $-NH_{2}$ forms Au-N bonds with gold to hold the nanoparticles. Meanwhile, for silicon substrates, (3-Aminopropyl) triethoxysilane (APTES) functionalization is more popular due to the stronger anchoring with the substrate (three bonds with silicon), while ethanolamine provides much denser coverage (one bond with silicon) [23,24]. An ethanolamine solution was prepared by mixing 3.3 mL of ethanolamine with 6.6 mL of DMSO and gently heated to 70 °C. Clean samples of silicon were then immersed in the solution and kept under vacuum condition inside a desiccator for 12 h. The amino- functionalized silicon samples were then removed and air dried.

In the final step, the amino-functionalized Si substrates were immersed in a gold nanoparticle solution for the self-assembly process at room temperature. Precisely, 5 mL of gold nanoparticle solution is mixed with 5 mL of 1× PBS solution and 40 mL of DI water. The substrates were kept immersed for different amounts of time (6 h, 11 h, 18 h, and 24 h) to control the gap-length between nanoparticles. After the desirable time, each substrate was removed, washed in DI water, and air dried.

For Raman spectroscopic measurements, methylene blue has been chosen as a Raman tag. Two solutions of MB are prepared of concentrations 1 mM and 1 µM, respectively. Around 10 µL of 1 µM MB is drop-casted on the Si substrates self-assembled with gold nanoparticles for different durations, and 1 mM MB is drop-casted over a clean silicon surface (no gold nanoparticles) for the control experiment. The droplets were allowed to dry under room temperature forming the coffee ring on the surface, and Raman spectroscopy was performed on the boundary of the resulting coffee ring.

### 2.3. Simulations

**Figure 1:**
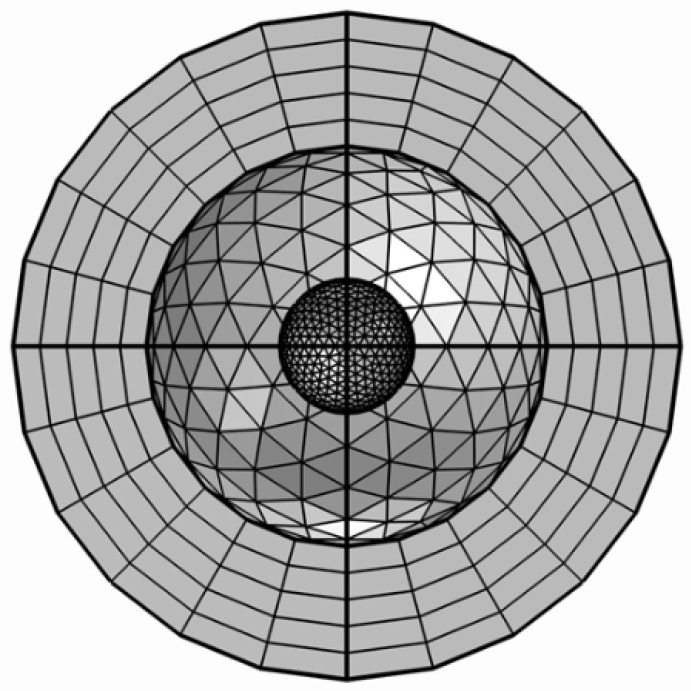
Geometry and mesh of single nanosphere with physical domain and perfectly matched layer.

Finite element modeling (FEM) of nanoparticles was performed using the COMSOL Multiphysics 5.0 wave optics module [18]. The model is developed by creating a spherical geometry of nanometer radius, as shown in Figure 1. Meshing related to other configurations is presented in Figure S1 in the Supplementary Information. In order to truncate the simulation physical domain, a perfectly matched layer (PML) is introduced, and a scattering boundary condition is applied on the inner surface of the layer shell. The perfectly matched layer is commonly used in radio frequency (RF) models as a boundary condition which provides a reflectionless interface for the outgoing wave at all incident angles. A background wave is defined as a transverse magnetic (TM) polarized wave with traveling in the positive x-direction, with the electric field polarized along the z-direction and unit amplitude ($E_{0z}=1\;V/m$), as shown in Equation (1). Amplitudes in other directions are considered to be zero.

(1) $$\vec{E}=E_{0z}\;exp(-jK_{0}x)\hat{z}$$

Here, $K_{0}$ is the wave number in free space. Simulations were performed over isolated nanospheres and multiple nanosphere systems in the wavelength domain. The numerical model in the COMSOL Multiphysics wave optics module solves the Maxwell’s equation with respect to the scattered electric field $E_{sca}$, as given in Equation (2).

(2) $$\nabla \times \left[\frac{1}{\mu_{r}}\left(\nabla \times E_{sca}\right)\right]-K_{0}^{2}\left[\left(\varepsilon_{r}-\frac{j\sigma }{\omega \varepsilon_{0}}\right)\right]E_{sca}=0$$

Here, $\mu_{r}$ is the relative permeability and $\varepsilon_{r}$ is the relative permittivity of the medium, σ is the conductivity of the material, ω is the angular frequency and $\varepsilon_{0}$ is the permittivity of free space. Materials were defined for each respective geometric domain. For the spherical domain around the nanosphere(s), the medium is defined as air with a real refractive index (n) of 1 and an imaginary refractive index (k) of 0. For the nanosphere medium, gold and silver were defined. Since the complex refractive index of noble metals varies with the incident radiation wavelength, a refractive index model is required to be defined. For our simulations, we used the Brendel-Bormann model of refractive indices for Au and Ag at different wavelengths, as described in Rakić et al. (1998) [25] and predefined in the material library of COMSOL Multiphysics.

## 3. Results and Discussion

### 3.1. Near Field Simulations and Optimization of Nanoparticle Systems

The enhancement factor of a nanoparticle-based SERS system will depend on the size of the nanoparticles and the gap between them. For this study, the nanoparticles are assumed to be spherical (nanospheres). For maximum electric field enhancement, the localized plasmons are required to resonate with the incident wavelength and the resonating frequency of a nanosphere depends on its radius. Again, the gap length between two nanospheres also influences the extent of dipole coupling, thereby effecting the enhancement factor of the SERS hotspot. The direction of polarization with respect to the orientation or respective position of nanospheres on a substrate can also effect the enhancement factor. It can be generally deduced that increasing the nanosphere radius in a multi-nanosphere system, while simultaneously decreasing the gap between them, can increase the enhancement factor of that respective hotspot. However, considering a practical sensor with an array of nanospheres, such an increase in nanosphere size will, on the other hand, decrease the total number of hotspots per unit area, which can effectively decrease the overall enhancement factor. Thus, the simulation of multiple nanosphere systems, varying nanosphere radius, the gap between two nanospheres, and their orientation with respect to polarization direction, is crucial to understanding their altogether effect and the respective trade-offs required to be made for the design of a SERS biosensing platform of maximum enhancement factor and, therefore, maximum sensitivity.

The enhancement factor (EF) of the SERS effect can generally be defined as how much the Raman signal can be amplified [26]. In most single molecule SERS studies [26,27,28,29], the single molecule enhancement factor can be defined as the fourth power of normalized maximum local electric field. Despite its over-simplification, the fourth-power rule of enhancement factor has been widely accepted in the field of single molecule SERS, and is suitable for the calculation of simulated enhancement factor in this study of hotspots of different multi-nanosphere systems. Since we defined a background wave of unity amplitude, the fourth power of local electric field will suffice for the enhancement factor calculations. When considering an array of nanospheres (described in Section 3.2—near field simulations and optimization of periodic nanoparticle array), a calculation of the cumulative effect of all hotspots is required, for which we defined an average enhancement factor [30] as an integration of single molecule enhancement factor over a unit area (Equation (3)). For analytical purposes (described in Section 3.4—Raman Spectroscopy with Raman Probe molecule), analytical enhancement factor (also known as experimental enhancement factor given in Equation (4)) can be defined as the ratio of the Raman intensity obtained from a SERS platform for a certain tag molecule with respect to Raman intensity obtained from its non-SERS counterpart, normalized over concentration [26,31,32,33].

**Figure 2:**
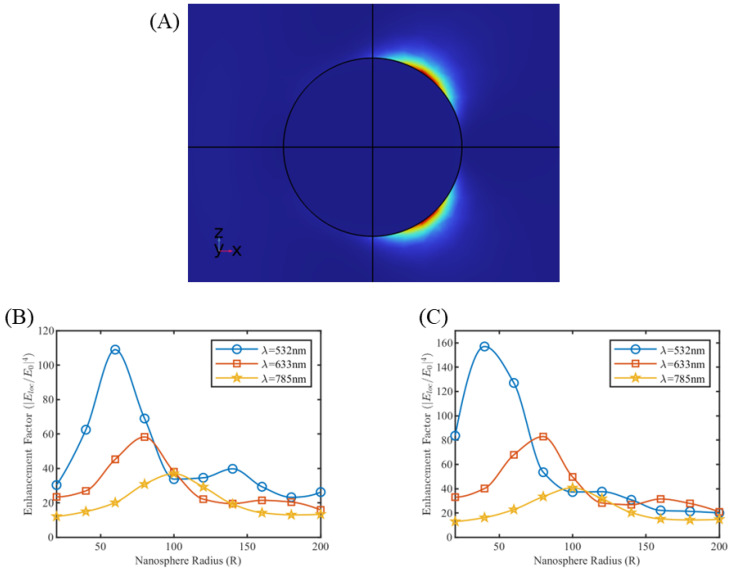
(**A**) Electrical field around a single nanosphere. Enhancement factor plots at different Raman wavelengths for single nanosphere of (**B**) silver and (**C**) gold.

Simulations were carried out for single silver and gold nanospheres of different size, bounded with the perfectly matched layer in the physical domain. Raman wavelengths of 532 nm, 633 nm, and 785 nm were selected for our simulations. For silver nanosphere, a maximum enhancement factor of 109 is calculated for a radius of 60 nm at 532 nm wavelength. As shown in Figure 2B, the enhancement factor peaks at different values of radius for different wavelengths, demonstrating the resonance effect of surface plasmons. Also, the value of peak enhancement factor decreases for increasing Raman wavelengths, suggesting that 532 nm may be the best wavelength to be applied for isolated silver nanoparticle. Similar results were obtained for single gold nanosphere simulation at different Raman wavelengths as shown in Figure 2C.

Some trends of simulation results in Figure 2 may not be according to general practice in the field of SERS. For example, in practice, silver nanoparticles are preferred over gold nanoparticles due to their higher EF [34], while from our simulations, gold nanoparticles are shown to have a slightly higher EF than silver nanoparticles. The gold nanoparticles show a higher EF at 633 nm than at 532 nm [35,36,37,38], however, our simulations in Figure 2 show the opposite. Both this reversal of trend can be explained by emphasizing the importance of plasmonic coupling. While in a practical setup, nanoparticles are assembled in multiple nanospheres systems, where the EF contribution coming from individual nanoparticles are fairly dominated (in the range of $10^{8}$) by the contributions from their respective coupling. In the subsequent sections, we performed simulations over multiple nanosphere systems, which have been seen to normalize the trend and minimize the discrepancy between the simulated results and general practical observations.

**Figure 3:**
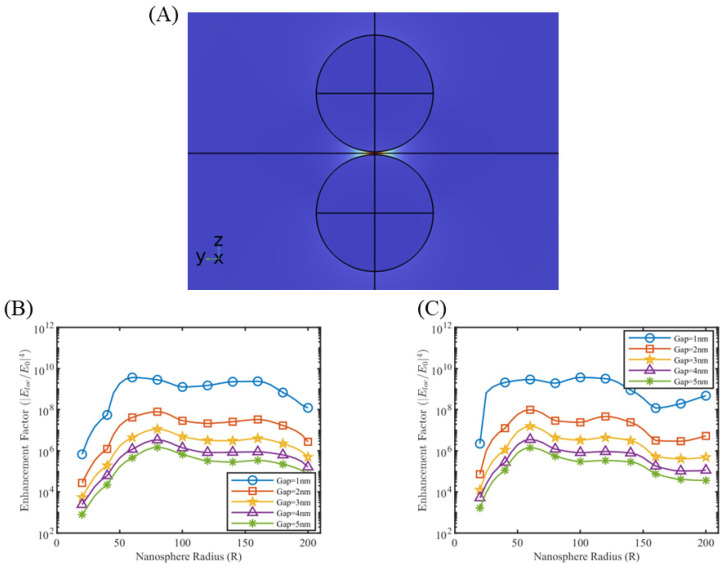
(**A**) Electric field of a nanodimer system. Enhancement factor plots of nanodimer system (**B**) gold at λ = 785 nm and (**C**) silver at λ = 633 nm.

A single nanosphere does not generate much enhancement factor (range ~$10^{2}$) to have practical applications. Simulations were extended to multiple nanosphere systems, to include the effect of dipole coupling. The geometry of the nanosphere dimer is defined such that the common axis aligns with the polarization (z-direction) of the background E-field (Figure 3A). Simulations over nanosphere dimers for different radii and gap lengths demonstrated the effect of SERS hotspot with enhancement factors in the range of $10^{6}–10^{9}$. Selected simulation results for silver and gold nanosphere dimer for wavelengths 633 nm and 785 nm, respectively, are shown in Figure 3B,C, and the rest of the simulation results are presented in Figures S2 and S3 of the Supplementary Information. As indicated in Figure 3B,C, the effective dipole coupling decreases with an increase in gap length, which decreases the enhancement factor. A maximum enhancement factor of $\sim 10^{9}$ occurred in case of silver nanodimers for a gap length of 1 nm over a radius range of 40–120 nm. Similarly for gold nanodimers, the maximum enhancement factor reached $\sim 10^{9}$ for a gap length of 1 nm over a radius range of 60–140 nm.

**Figure 4:**
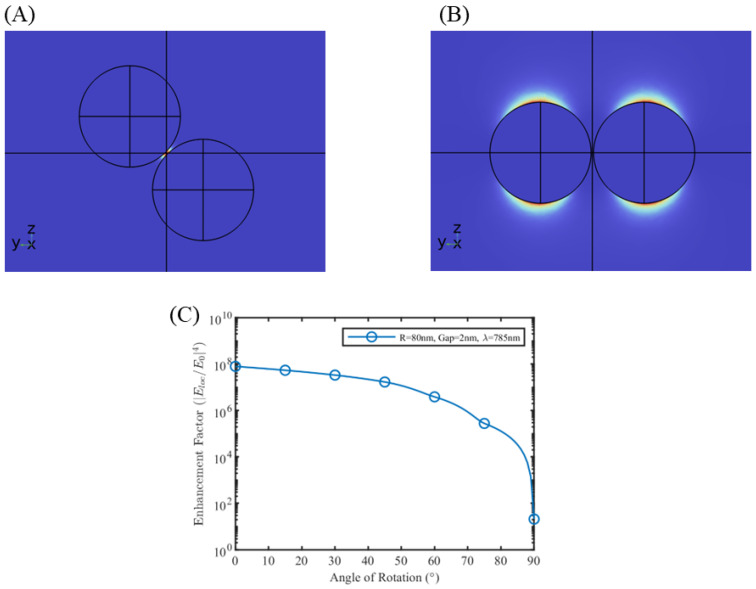
Electrical field of the nanodimer system at orientation (**A**) 45° and (**B**) 90° with respect to the direction of incident polarization. (**C**) Enhancement factor variation with orientation for gold nanodimers of radius 80 nm, gap 2 nm and λ = 785 nm.

**Figure 5:**
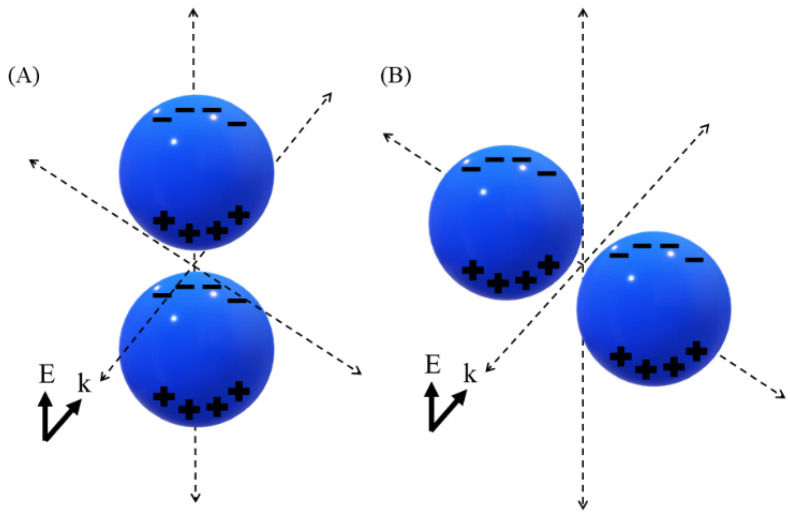
Polarization charge of nanodimer system at different orientations: (**A**) perfectly aligned with the direction of polarization and (**B**) perpendicular alignment with the direction of polarization.

Orientation of the nanodimers with the polarization of the background radiation has a significant effect on the enhancement factor. Maximum enhancement factor is observed when the nanodimers are aligned with the direction of polarization (z-axis in this case). When the dimers are rotated in the y–z plane (Figure 4A,B), the enhancement factor of the hotspot decreases exponentially, with complete loss of the hotspot observed at perpendicular alignment (Figure 4B). For a single nanosphere, we observed that the area of maximum localized electric field occurs in the plane of electric field polarization, and when two such areas come to close proximity, as in the case of nanodimers, the effective dipolar coupling of surface plasmons results in a very high enhancement of the local electric field. With the rotation of the dimer system, the effective plasmon coupling decreases, with no coupling at perpendicular alignment. As per hybridization theory [33], a high dipolar electric field is generated when polarization charges of opposite types on the nanosphere surface come close to each other in the case of perfect alignment, and no such field is generated for perpendicular alignment as shown in Figure 5. Such a phenomenon of polarization-dependent SERS is an important point for SERS biosensing surfaces with a regular array of nanostructures, as the alignment of polarization with the array direction is required to achieve maximum enhancement.

**Figure 6:**
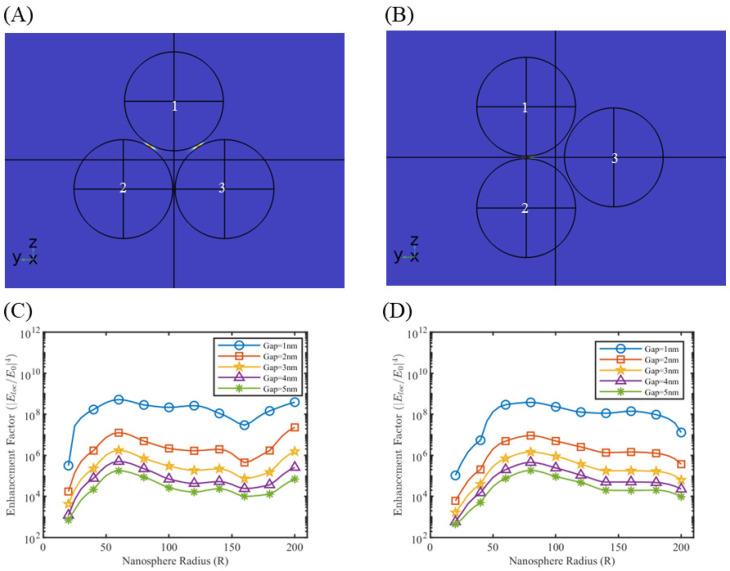
Electrical field of nanotrimer system for different orientations (**A**) nanotrimer orientation A and (**B**) nanotrimer orientation B. Here polarization is in the positive z-direction. Enhancement factor plots for nanotrimer system orientation A for (**C**) silver at λ = 633 nm and (**D**) gold at λ = 785 nm.

**Figure 7:**
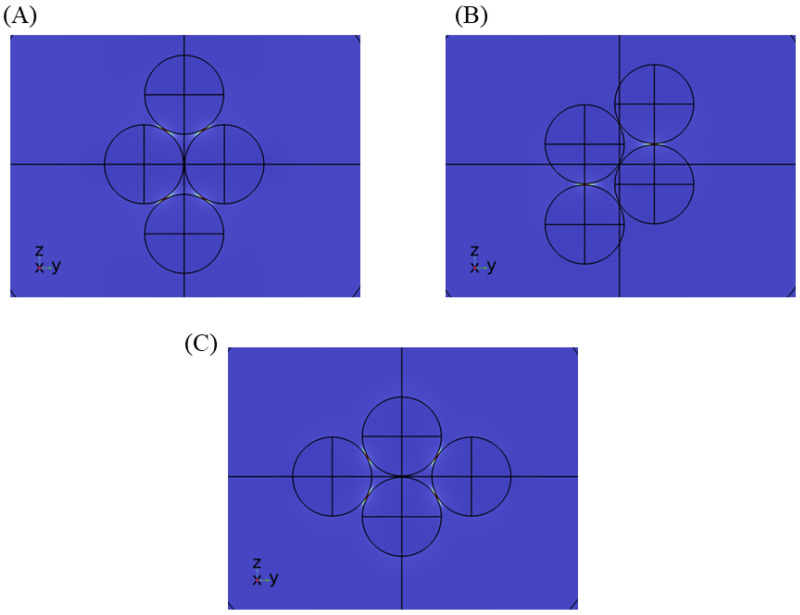
Electric field of nanotetramer systems at different orientations (**A**–**C**). The direction of polarization is positive z.

**Figure 8:**
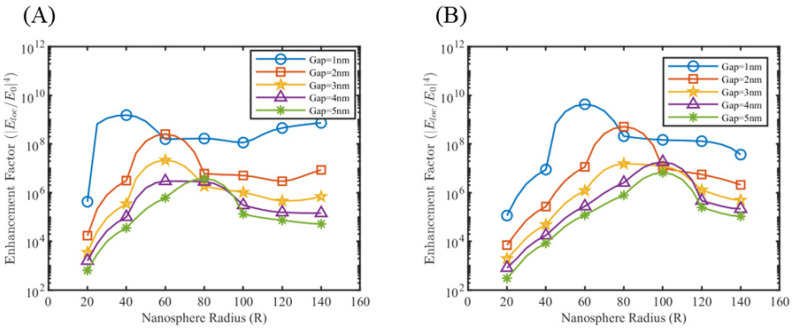
Enhancement factor plots for nanotetramer system orientation C for (**A**) silver at λ = 633 nm and (**B**) gold at λ = 785 nm.

Multiple nanosphere systems with three (trimers) and four (tetramers) nanospheres were simulated, similarly taking care of the different orientations possible with respect to the direction of background wave polarization. For nanotrimers, three nanospheres were placed on the three vertices of an equilateral triangle, maintaining the same gap length between them. As shown in Figure 6, two orientations are possible in this case. Figure 6A shows the orientation where there is a partial plasmonic coupling between nanosphere 1–2 and nanosphere 1–3, but no coupling between nanosphere 2–3. We call this orientation the nanotrimer orientation A. Figure 6B can be obtained by simply rotating the nanotrimer orientation A anti-clockwise in the z–y plane by 30°. In this case, there is a full plasmonic coupling between nanosphere 1–2 and negligible coupling with nanosphere 3. We call this orientation the nanotrimer orientation B. Enhancement factor plots for silver and gold nanotrimers at 633 nm and 785 nm at nanotrimer orientation A are shown in Figure 6C,D, with the rest of the simulation results depicted in Figures S4–S7 of the Supplementary Information. Nanosphere tetramers were modeled by placing four nanospheres on the vertices of a rhombus. This geometry allows the formation of a maximum of five possible hotspot locations. In the case of nanotetramers, three orientations (nanotetramer orientation A, B, C) are possible, as shown in Figure 7, and accordingly two to five hotspot regions can activate at a time. The enhancement factor plots for selected cases are shown in Figure 8, with the rest of the simulation result included in Figures S8–S11 of the Supplementary Information. Similar to nanodimers, the maximum enhancement factor is in the range of $10^{5}–10^{9}$, despite adding more nanospheres in the system. This is due to dipolar misalignment with the direction of polarization at a particular orientation, which does not allow for the full plasmonic coupling of all the nanospheres. Nonetheless, a nanotrimers and nanotetramer system generate multiple hotspots which should contribute to better sensitivity of the overall sensor system.

**Figure 9:**
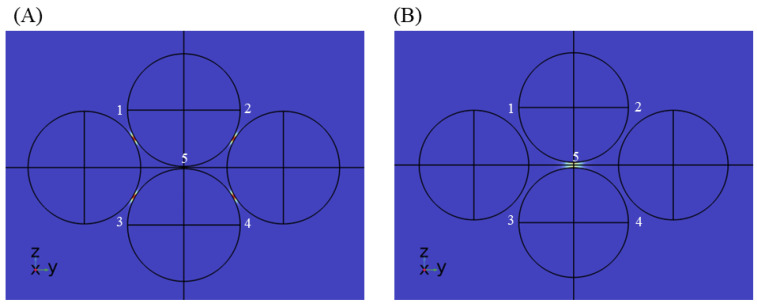
Nanotetramer system at orientation C, showing the phenomenon of hotspot switching. (**A**) Hotspot 1–4 are on, hotspot 5 is off for silver nanotetramer radius 100 nm, gap of 4 nm and λ = 633 nm. (**B**) Hotspot 1–4 are off, hotspot 5 is on for silver nanotetramer radius 40 nm, gap of 4 nm and λ = 633 nm.

**Figure 10:**
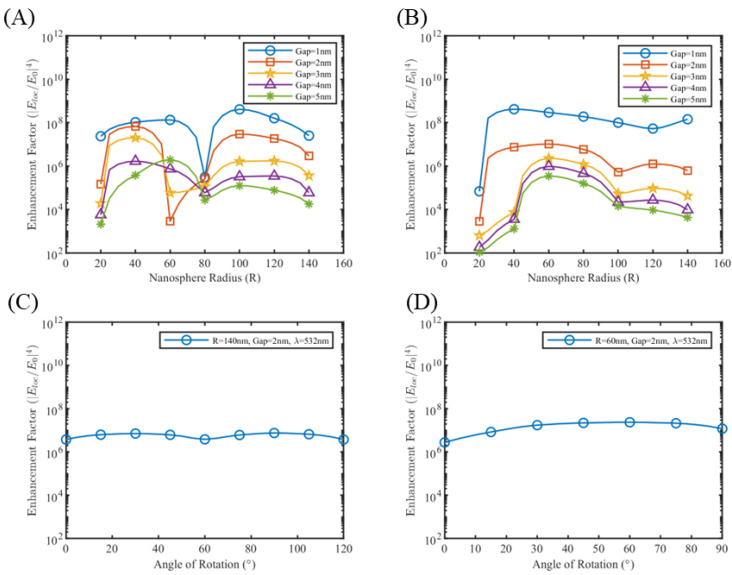
Enhancement factor plot of (**A**) hotspot 5 and (**B**) hotspot 1 for silver nanotetramer at λ = 532 nm. Variation of enhancement factor with orientation of silver (**C**) nanotrimer of radius 140 nm, gap 2 nm at λ = 532 nm and (**D**) nanotetramer of radius 60 nm, gap 2 nm at λ = 532 nm.

Hotspot 5 (Figure 9) gets activated for particular combinations of nanosphere size, gap length, and wavelength. In order to understand the hotspot switching, simulations were performed with silver medium, probing into individual hotspot enhancement factors. The enhancement factor for hotspot 1 and hotspot 5 is probed for all combinations of nanosphere radius, gap lengths, and wavelengths. Figure 10A shows that hotspot 5, or the hotspot between two nanospheres perfectly aligned with the polarization direction, has a tendency to switch off for a particular radius value. As for 532 nm wavelength, this switch-off happens for a radius of 60 nm or 80 nm depending on gap length. The enhancement factor for hotspot 1, on the other hand, remains fairly constant for these radius values. Similar results were obtained for 633 nm and 785 nm wavelengths, where the radius window of switch-off shifted to larger radius values (see Figures S14 and S15 in Supplementary Information). This switch-off could be the result of the multiple resonance pattern between the nanosphere system, and a better explanation requires understanding the plasmonic hybridization between multiple nanospheres in this system. Similar switch-off patterns were observed for nanotrimers system orientation B as well (see Figures S12 and S13 in Supplementary Information), however, the extent of enhancement factor reduction is much less than that of nanotetramers, suggesting that the nanospheres on the sides influences the switching off. We also performed an orientation sweep study on both the nanotrimers and nanotetramers systems, discovering that the hotspot switching makes the enhancement factor of the systems fairly polarization-independent, as shown in Figure 10C,D.

### 3.2. Near Field Simulations and Optimization of Periodic Nanoparticle Array

**Figure 11:**
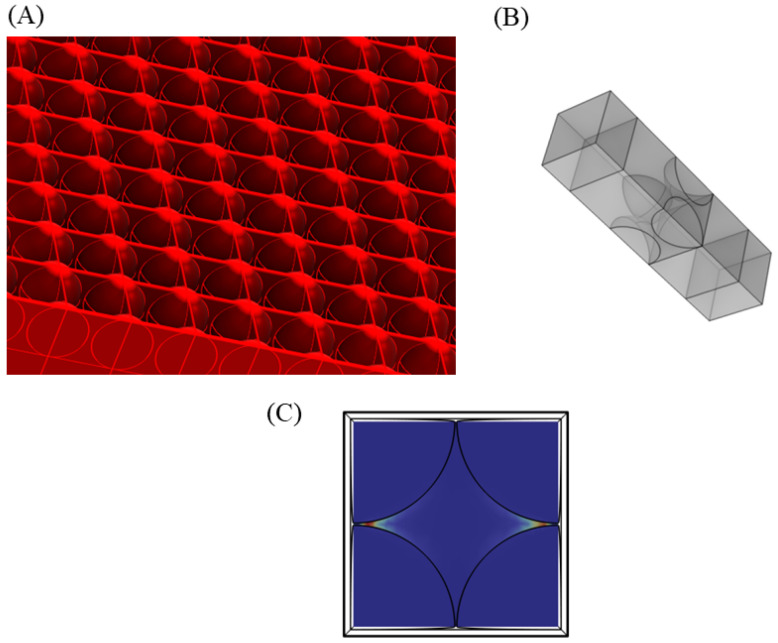
(**A**) A three-dimensional view of a periodic array of nanospheres. (**B**) Unit cell of the periodic nanosphere array. (**C**) Enhancement factor of the unit cell showing an effective domain of one nanosphere only.

Finite element modeling of an array of nanospheres on a selected substrate provides a comprehensive understanding of the biosensing system. A finite array of nanospheres can be computationally demanding, which limits the number of nanoparticles that can be simulated. Periodic boundary conditions in COMSOL allow one to simulate periodic structures by computing only over one unit cell. The periodic unit cell of an array of nanospheres is shown in Figure 11, which effectively reduces the computation to one nanosphere only. Meshing details are also provided in Figure S16 in Supplementary Information. In the model, we described the upper medium as water with a real refractive index (n) of 1.33 and an imaginary refractive index (k) of 0. The lower substrate is defined as Silicon with a predefined refractive index model according to Green et al. (2008) [39].

In order to ensure proper compilation of the model, one needs to ensure that the free triangular mesh on opposite faces with periodic boundary conditions applied are exactly the same. Mesh of one face is copied to the opposite one to ensure proper application of the periodic boundary condition. PML layers were defined on both the ends of the model to ensure proper truncation of the physical domain. Periodic boundary conditions with floquet periodicity are applied on each opposite wall of the physical domain. Instead of a background wave, port boundary conditions are required to be defined in this case. The input port with an intensity of $0.001\;mW/\mu m^{2}$ is defined, emitting a linearly polarized wave traveling in x-direction and polarization in z-direction. An average enhancement factor is evaluated from the simulations which are defined in Equation (3) [30].

(3) $${EF}_{av}=\frac{1}{A_{0}}\iint {\left|\frac{E_{loc}}{E_{0}}\right|}^{4}\;dA$$

**Figure 12:**
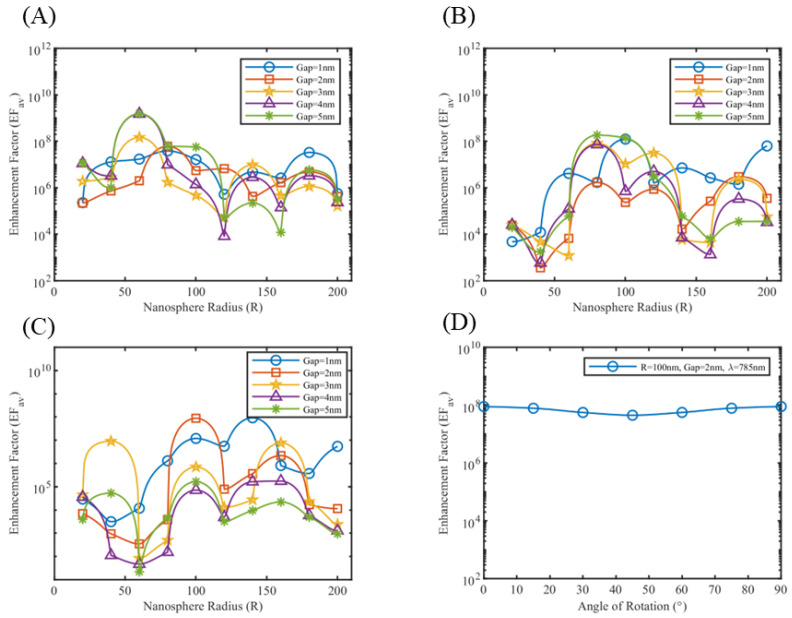
Average enhancement factor plot of periodic silver nanospheres at (**A**) λ = 532 nm, (**B**) λ = 633 nm and for periodic gold nanospheres at (**C**) λ = 785 nm. (**D**) Variation of average enhancement factor with orientation of a periodic gold nanosphere array of radius 100 nm, gap 2 nm at λ = 785 nm.

Here, $A_{0}$ is the area of the unit cell of silicon substrate, undecorated with nanoparticles. The enhancement factor plots for silver nanospheres at 532 nm and 633 nm wavelengths and gold nanospheres at 785 nm wavelength are shown in Figure 12A–C, with the rest of the simulations included in Figures S17 and S18 of the Supplementary Information.

As shown in Figure 11C, only two of four hotspots are activated, which are aligned with the direction of polarization. The enhancement factor by definition provides the average enhancement of the dipolar coupling between two metallic nanospheres, and the hotspot between metal and semiconductor junctions. This results in a key difference between the enhancement factor plots of free nanosphere models vis-à-vis enhancement factor plots in Figure 12. The average enhancement factor provides a comprehensive picture for SERS biosensing platform simulations. Polarization-dependent enhancement factor for periodic nanoparticle structure is studied for gold nanospheres at 785 nm. As the direction of polarization is rotated from 0° to 90° (i.e., from z-direction to y-direction), the enhancement factor of hotspots aligned along the z-direction decreases, while the enhancement factor of hotspots aligned along the y-direction increases. This keeps the average enhancement factor over the unit cell fairly constant, as shown in Figure 12D.

### 3.3. Limitation of the Model

The above-described model, with isolated noble metal nanospheres or a periodic array of noble metal nanospheres, solves the Maxwell’s equation for scattering E-field, and can be applied for various applications including SERS biosensing. However, the current model does not include the charge transfer between two metallic nanospheres via quantum tunneling and the charge transfer in the metal–semiconductor junction. Charge transfer via quantum tunneling in the metal nanogaps can decrease the overall SERS enhancement factor [40], which cannot be tapped into with this current model. For this reason, the nanogaps between metal nanospheres in the simulations are limited to a minimum value of 1 nm only. Nanogaps less than 1 nm will have the significant effect of quantum tunneling, thereby increasing the error of our simulated results. A better model, including the quantum tunneling effects with Maxwell’s equations, is in the scope of our future research.

### 3.4. Raman Spectroscopy with Raman Probe Molecule

The enhancement factor of the self-assembled gold nanoparticles over Si substrate is measured by performing Raman spectroscopy over the surface with a desired Raman tag. For our experiments, methylene blue (MB) was chosen as the Raman tag. For analytical enhancement factor measurements, a lower concentration of MB (1 µM) was dried over the prepared SERS substrate (Si substrate with self-assembled gold nanoparticles described in Section 2.2), and a higher concentration of MB (1 mM) was dried over a bare Si substrate for experimental control.

Analytical (or Experimental) Enhancement factor can be calculated according to Equation (4) [33].

(4) EFexp=ISERSCSERSIRamanCRaman

Here, $I_{SERS}$ is the characteristic peak intensity of the tag molecule obtained from the modified SERS surface at a concentration of $C_{SERS}$, and $I_{Raman}$ is the intensity of the same characteristic peak of the same tag molecule obtained from an unmodified surface at a concentration of $C_{Raman}$.

**Figure 13:**
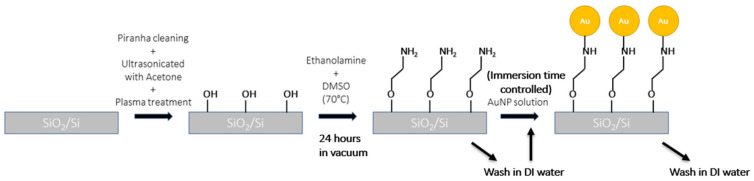
Proper functionalization scheme of gold nanoparticles by aminating the Si surface.

**Figure 14:**
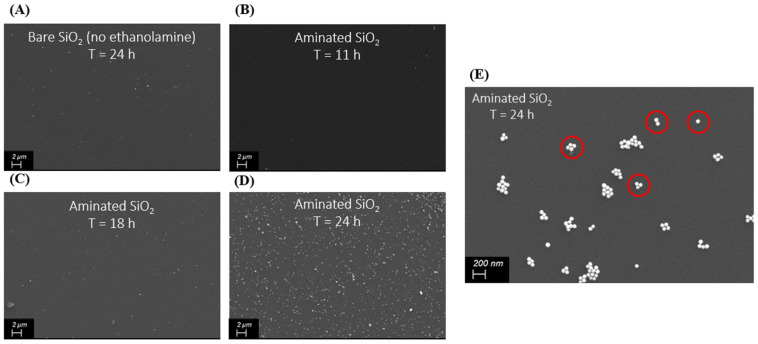
Scanning electron microscopy images of gold nanoparticles self-assembling over (**A**) bare Si substrate without any amination after 24 h immersion, (**B**) aminated Si substrate after 11 h immersion, (**C**) aminated Si substrate after 18 h immersion and (**D**) aminated Si substrate after 24 h immersion (**E**) Higher magnified image of gold nanoparticles self-assembling over aminated Si substrate after 24 h immersion showing the formation of different nanoparticle systems—single nanosphere, nanodimer, nanotrimer, and nanotetramer (as highlighted by red circles).

The surface of the Si substrates functionalized with gold nanoparticles (Figure 13) has been imaged using scanning electron microscopy (Zeiss Ultra FESEM) and shown in Figure 14. As expected in Figure 14A, without any proper amination, not a significant amount of gold nanoparticles adhered with the surface. After amination and proper immersion in the colloidal gold nanoparticle solution, a significant quantity of gold nanoparticles adhered to the surface, proportional to the immersion time, as shown in Figure 14B–D. We observed that this process of self-assembly lacks proper control for the precise placement of gold nanoparticles. The immersion time beyond 24 h started to produce multiple layers of gold nanoparticles on top of each other. Nonetheless, under higher magnification in Figure 14E, we can resolve the (apparently uncontrolled) formation of all the different nanosphere systems that have been considered for this simulation study—single nanosphere, nanodimer, nanotrimer, nanotetramer, and even more complex multi-nanoparticle systems. The average size of the nanoparticles was measured to be around 93.045 nm, with the average gap measured between these nanoparticles in individual systems around 5.38 nm. Unfortunately, probing each individual system for Raman measurements is a far more challenging experimental problem and is out of scope for this current study. The cumulative effect of all the multi-nanoparticle systems is reflected in the measured analytical EF using Raman spectroscopy.

**Figure 15:**
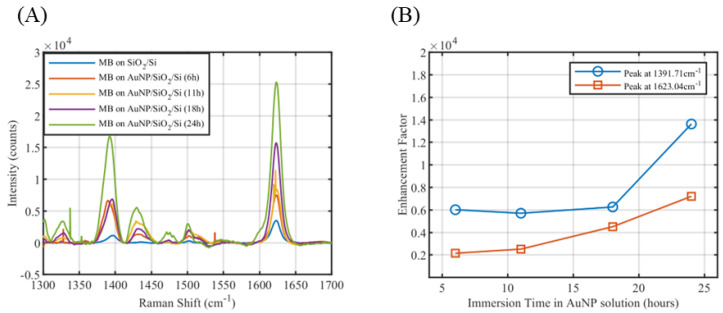
(**A**) Baseline corrected Raman spectrum of 1 mM methylene blue on bare SiO_2_ surface and 1 µM methylene blue on SiO_2_ surface self-assembled with gold nanoparticles at different immersion times. (**B**) Enhancement factor measured for two signature peaks of methylene blue at different immersion times in gold nanoparticle solution.

Since for an unmodified SiO_2_ surface, the Raman peak intensity of 1 µM MB could be quite less, we drop-casted 1 mM MB to determine $I_{Raman}$. Raman spectroscopy was performed using a Horiba Jobin Yvon HR800 LabRAM Raman Spectrometer, Pennsylvania, USA, over each surface at 633 nm wavelength. An acquisition time of 120 s with an accumulation of 2, grid size of 1800 gr/mm, and 10% intensity filter settings were applied for our Raman experiments. Initially, the Raman spectrum was auto-calibrated to $520.74\;{cm}^{-1}$ with a SiO_2_ surface. The baseline of each obtained Raman spectrum were corrected by subtracting a B-Spline interpolated baseline curve over 12 points in Origin [41]. The baseline-corrected Raman spectrums of 1 mM MB over SiO_2_ surface and 1 µM MB over self-assembled AuNP over SiO_2_ surface are shown in Figure 15A. Between $1300\;{cm}^{-1}$ and $1700\;{cm}^{-1}$, two characteristic peaks of methylene blue are obtained at $1391.71\;{cm}^{-1}$ and $1623.04\;{cm}^{-1}$, corresponding to the assigned band v(C−C) ring and $\alpha \left(C-H\right),$ respectively [42,43]. The enhancement factor plot is shown in Figure 15B. From the SEM imaging in Figure 14B,C, it can be observed that not a significant amount of nanoparticles were functionalized for an immersion time of 11 h and 18 h, which resulted in fewer gold nanoparticles with higher intermetallic gaps, and thus a much lower enhancement factor close to $0.6\times 10^{4}$. As expected, with a higher immersion time of 24 h, more gold nanoparticles were able to self-assemble over the surface, thereby increasing the total number of functionalized gold nanoparticles and decreasing the intermetallic nanogaps between them (Figure 14D). Accordingly, the enhancement factor increased to $1.3\times 10^{4}$. Correlating with our simulated results of periodic gold nanospheres on silicon substrate at a 633 nm wavelength (Figure S18b), we obtained a single molecule enhancement factor in the range of $10^{4}–10^{5}$ for nanoparticles of radius 50 nm for different gap lengths. This shows reasonable consistency between our complex SERS simulation and our simplified experiment.

The simple nature of our experiment does include certain biases to the measurements. Firstly, the uncontrolled functionalization of gold nanoparticles over the Si substrate does not imitate the regular periodic nature of the model and could contribute to the difference between the measured and calculated enhancement factors. Secondly, the quantum tunneling effect due to the metal–semiconductor junction of the gold–silicon functionalization gap, or in different areas of the substate where the inter-metallic gap could be less than 1 nm, can contribute to a lowering of the practically achieved enhancement factor. Despite such biases, single molecule enhancement factor calculation from some cases of our model correlates well with the analytical enhancement factor measurements, signifying its robustness and impact. Unfortunately, some important results from our simulations cannot be verified with this simple experimental study. Further complex experimental studies are required to confirm other aspects of the model, including isolated enhancement factors of different nanoparticle systems and the effect of incident wave polarization on them, with potential application for single molecule biosensing.

## 4. Conclusions

This study presents a comprehensive simulation study and simplified experimental work to understand the scattering properties and SERS enhancement factors of noble metal nanospheres. Our research highlights several key findings [44]:

The model addresses complete scattering E-field solutions via Maxwell’s equations for isolated noble metal nanospheres, different nanosphere systems such as nanodimer, nanotrimer, and nanotetramer, and periodic arrays of nanospheres to compute single-molecule enhancement factors. Meticulous evaluations were made for the enhancement factors for silver and gold nanospheres at various orientations (with respect to incident wave polarization) of different nanosphere systems, revealing their polarization-dependent behavior. Such polarization-dependent enhancement factor change revealed a very interesting and novel phenomenon of local hotspot switching in these regularly structured metallic nanosphere systems. This signifies the importance of incident wave polarization with respect to the orientation of multi-nanosphere systems for obtaining maximum sensitivity of nanoparticle-based SERS biosensors.

A simplified experimental work was performed with Raman spectroscopy using methylene blue (MB) as the Raman tag, where the analytical enhancement factor of self-assembled gold nanoparticles on silicon substrates was examined. With higher immersion time during the functionalization process, a significant number of gold nanoparticles were functionalized over aminated silicon substrate, forming an effective SERS platform with an analytical enhancement factor in the range of $10^{4}$. This correlated well with the single molecule enhancement factor in the range of $10^{4}–10^{5}$ calculated using our complex SERS model of periodic gold nanosphere array. Certain experimental biases such as quantum tunneling and irregularity of the nanoparticles could explain the reduction in experimentally achieved enhancement factors from the simulated one. Despite the simplified nature of the experiment, such correlation signifies the robustness of this model and its potential impact for nanoparticle-based SERS biosensor design.

Overall, this work provides a solid framework for further exploration into the effects of nanoscale phenomena on SERS, paving the way for improved biosensing technologies. Future research focusing on integrating quantum mechanical effects into this existing model can enhance predictive accuracy and experimental relevance.

## Data Availability

Data available on request from the Corresponding Author.

## Supplementary Materials

The following supporting information can be downloaded at: https://www.mdpi.com/article/10.3390/s25041165/s1.
