# Supplementary material for: Design and Optimization of a Gold and Silver Nanoparticle-Based SERS Biosensing Platform
Source: Sensors (Basel). 2025 Feb 14;25(4):1165. doi: 10.3390/s25041165 (PMC11859697; doi:10.3390/s25041165)
Supplement: Supplementary file 1 [file sensors-25-01165-s001.zip › sensors-3419511-supplementary.pdf]

Design and Optimization of a Gold and Silver Nanoparticle-Based SERS Biosensing Platform

Soumyadeep Saha[1,2], Manoj Sachdev[2] and Sushanta K. Mitra[1,*]

[1] *Micro and Nanoscale Transport Laboratory, Department of Mechanical & Mechatronics Engineering, Waterloo Institute for Nanotechnology, University of Waterloo, Waterloo, ON, N2L3G1, Canada.*

[2] *Department of Electrical & Computer Engineering, Waterloo Institute for Nanotechnology, University of Waterloo, Waterloo, ON, N2L 3G1, Canada.*

* *Corresponding Author Email: skmitra@uwaterloo.ca*

Supplementary Information

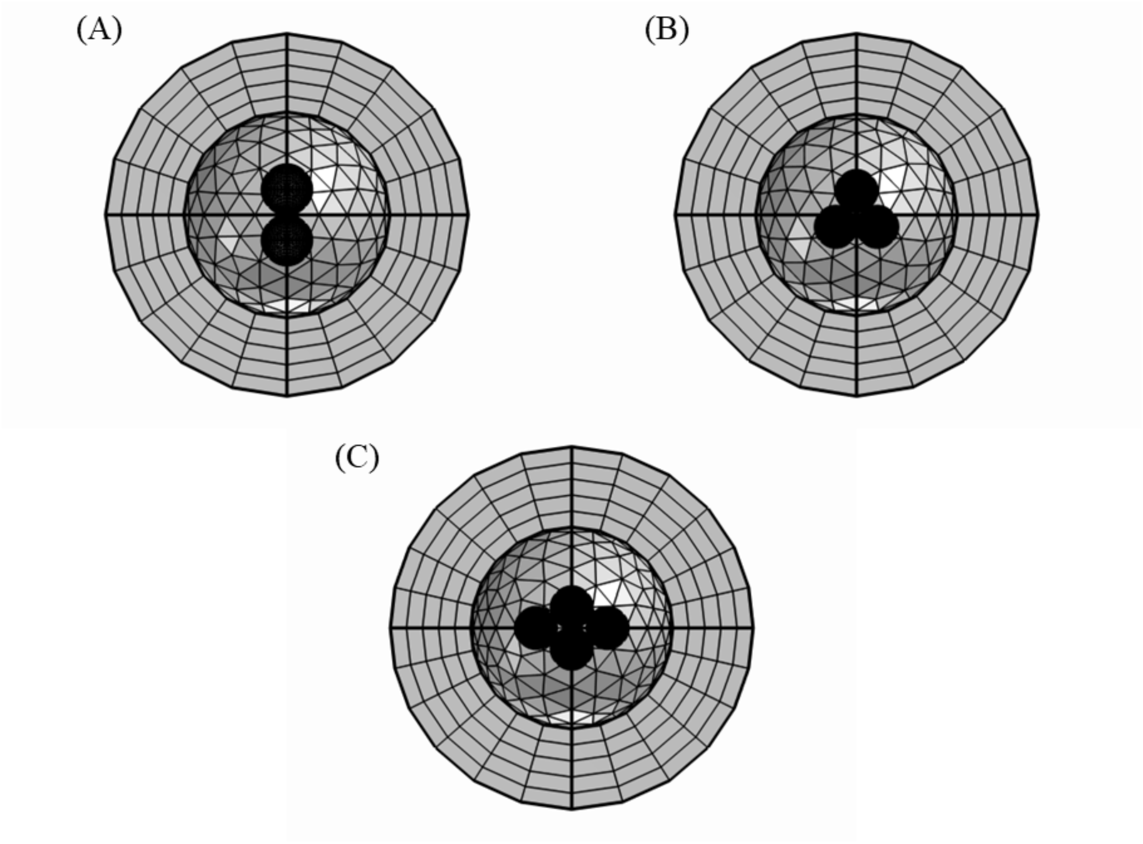

Figure S1: *Geometry and meshing of the multiple nanospheres system with the physical domain and perfectly matched layer (a) nanodimer (b) nanotrimers (c) nanotetramer.*

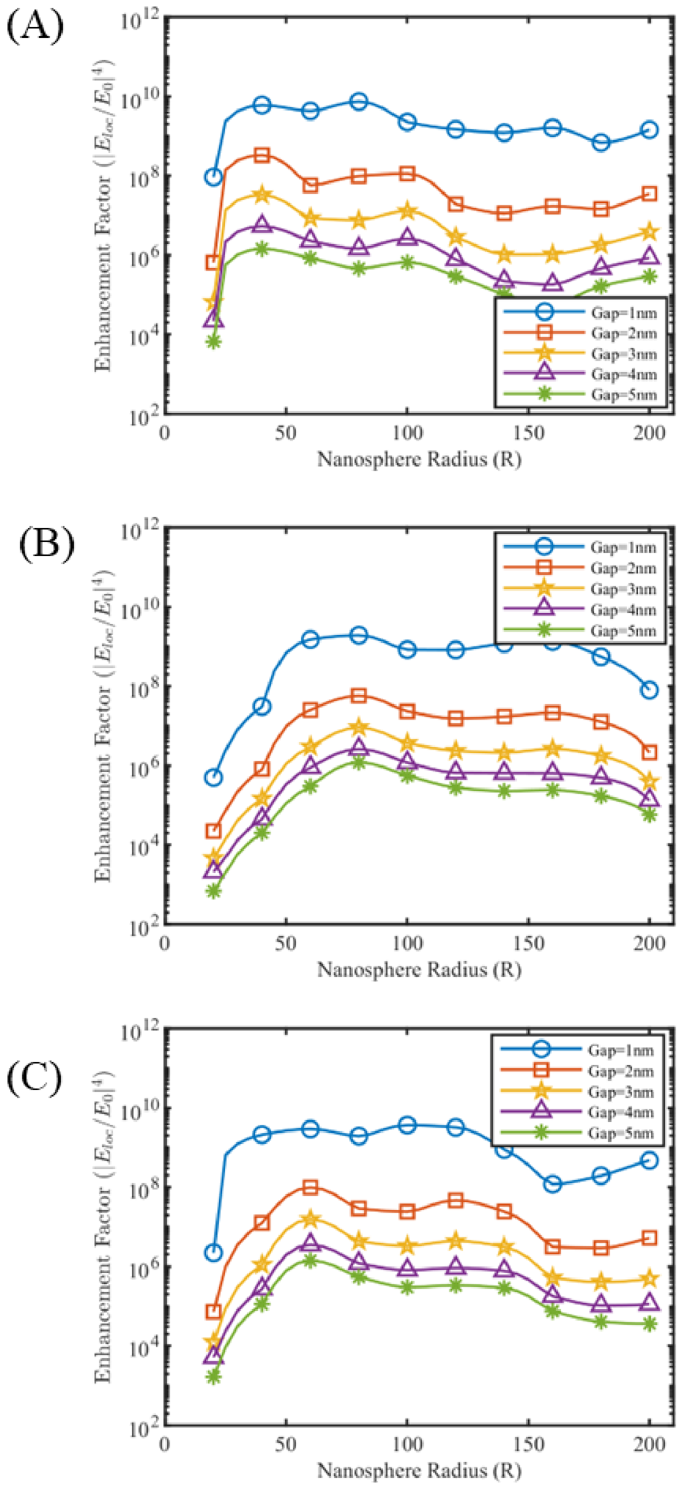

Figure S2: *Enhancement factor plots of silver nanodimer at wavelength (a) 532nm (b) 633nm and (c) 785nm.*

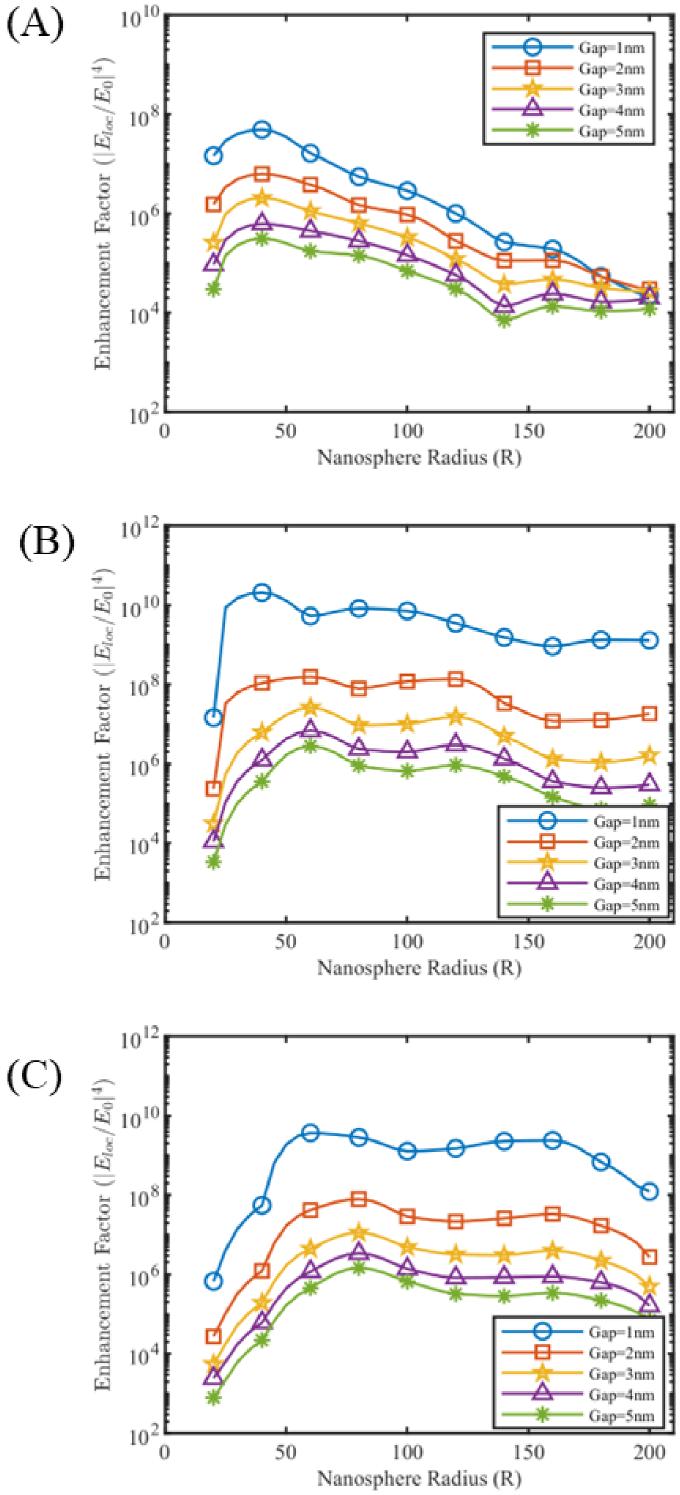

Figure S3: *Enhancement factor plots of gold nanodimer at wavelength (a) 532nm (b) 633nm and (c) 785nm.*

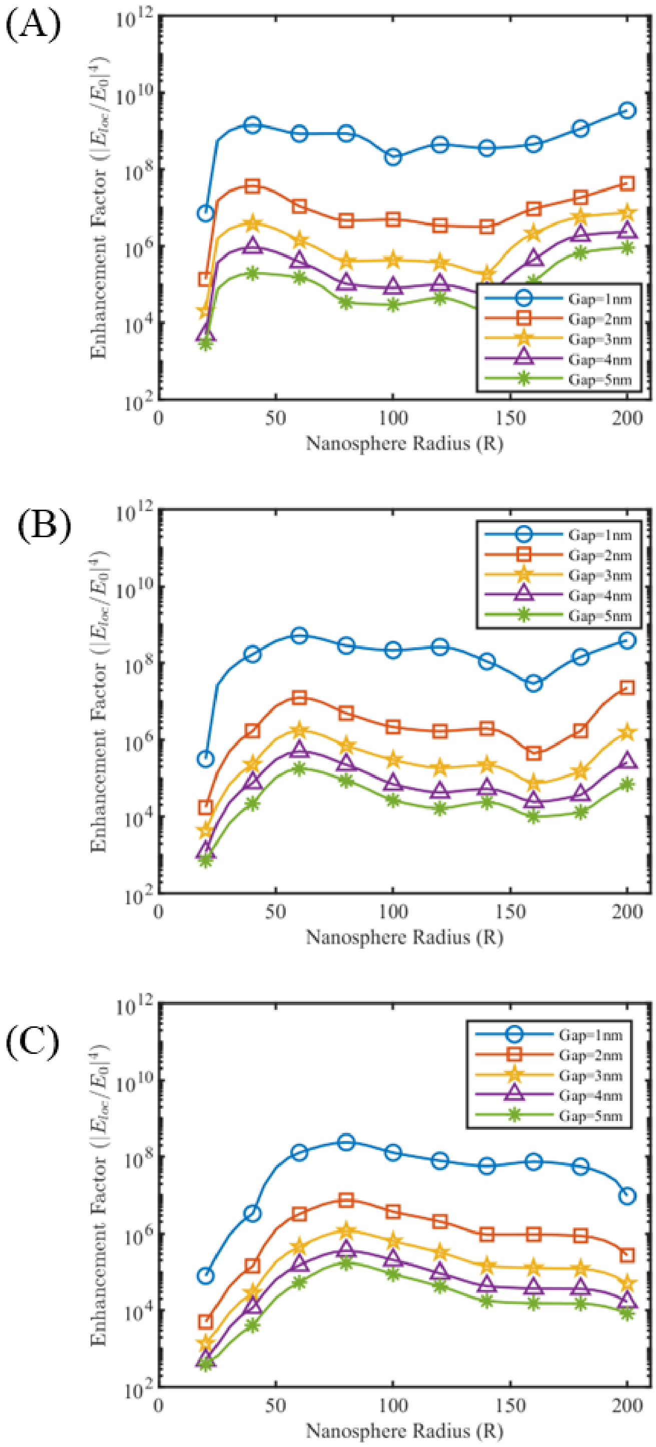

Figure S4: *Enhancement factor plots of silver nanotrimer orientation A at wavelength (a) 532nm (b) 633nm and (c) 785nm.*

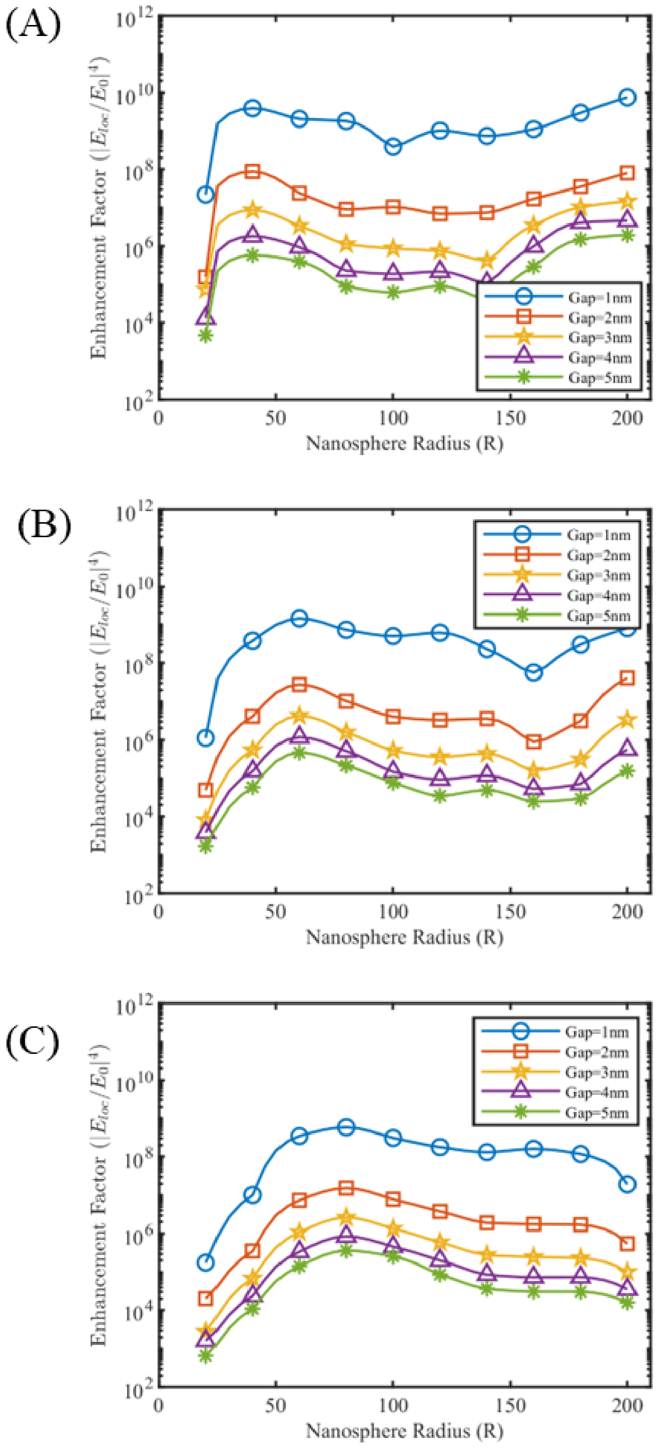

Figure S5: *Enhancement factor plots of silver nanotrimer orientation B at wavelength (a) 532nm (b) 633nm and (c) 785nm.*

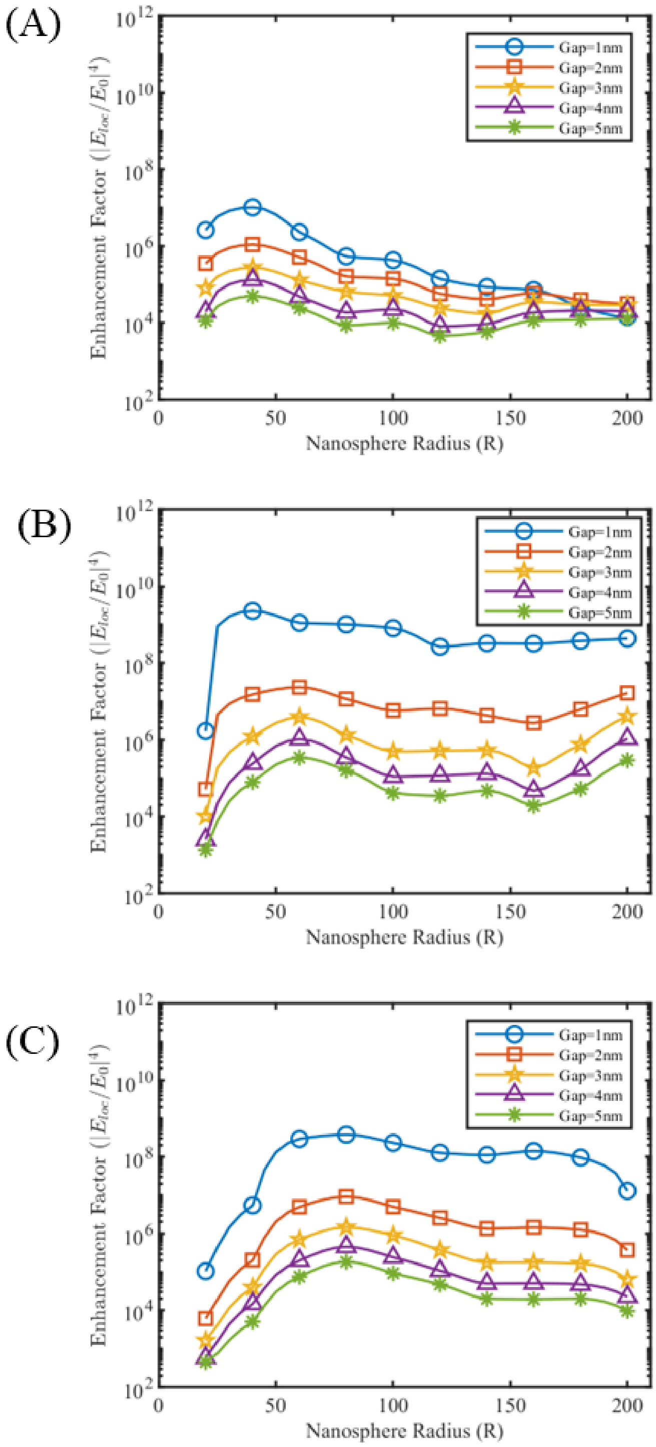

Figure S6: *Enhancement factor plots of gold nanotrimer orientation A at wavelength (a) 532nm (b) 633nm and (c) 785nm.*

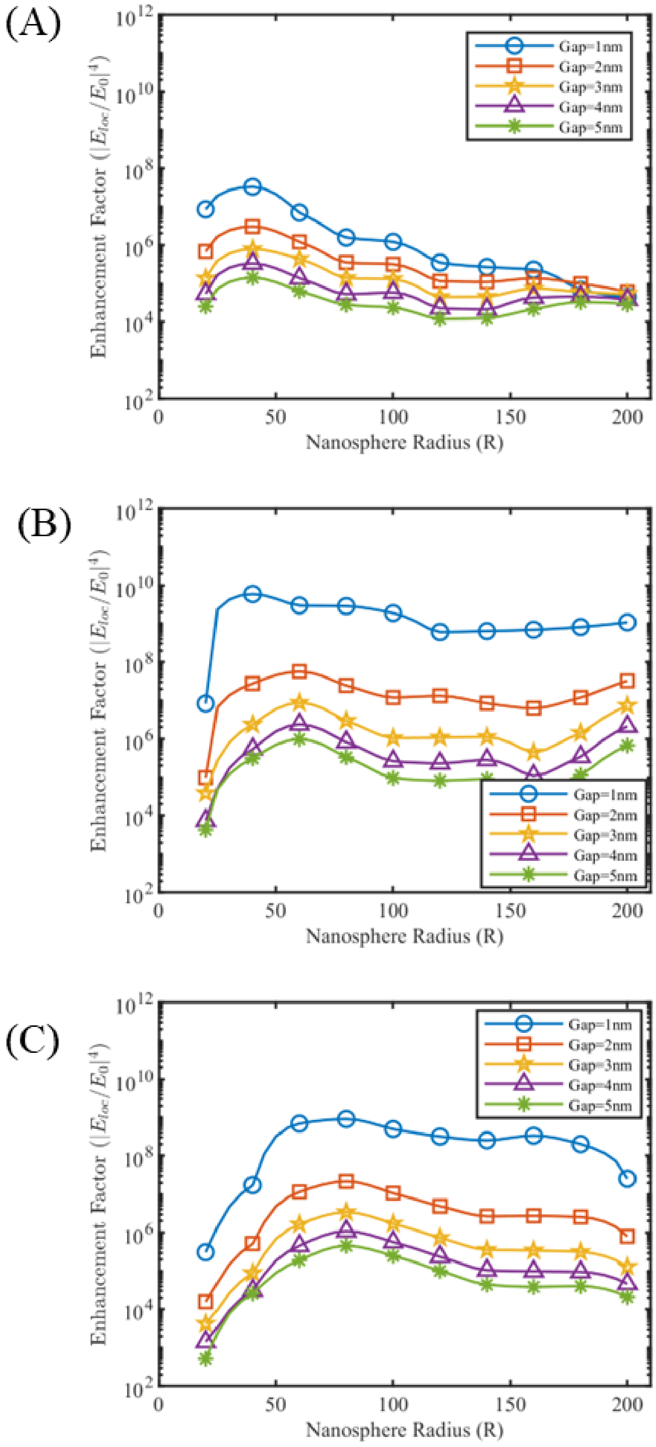

Figure S7: *Enhancement factor plots of gold nanotrimer orientation B at wavelength (a) 532nm (b) 633nm and (c) 785nm.*

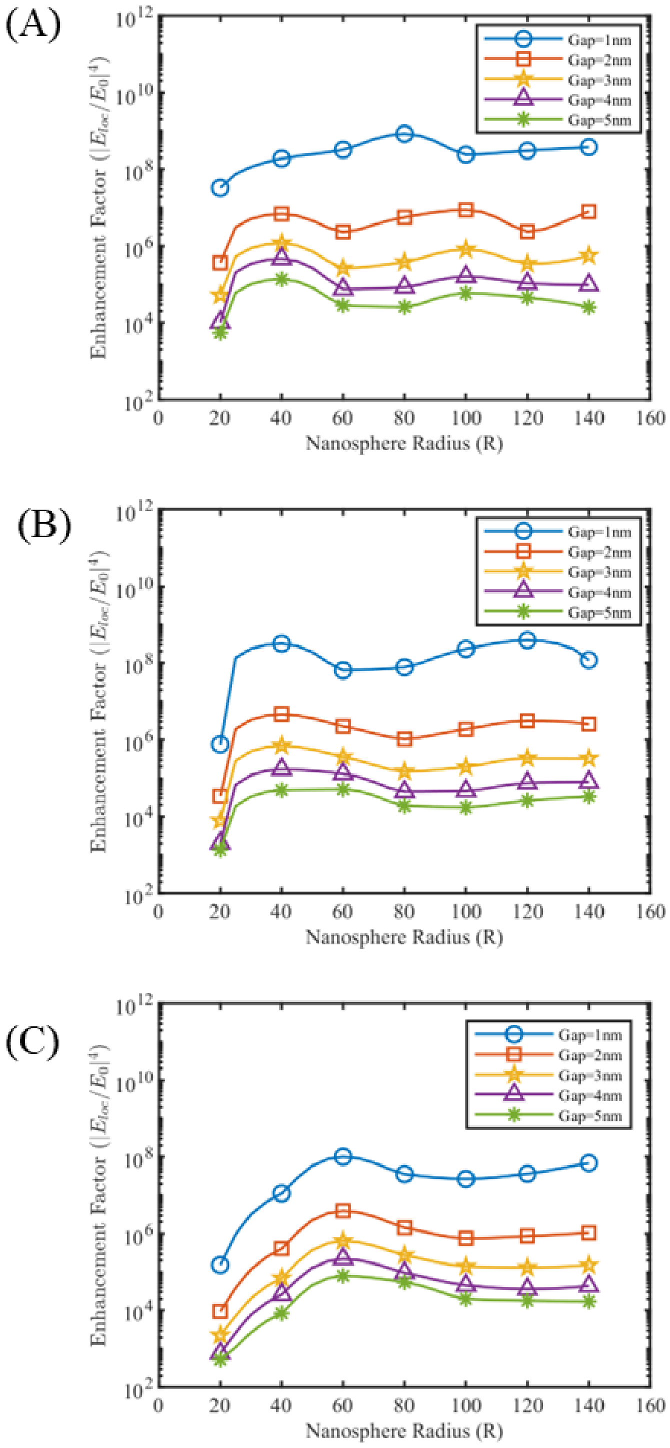

Figure S8: *Enhancement factor plots of silver nanotetramer orientation A at wavelength (a) 532nm (b) 633nm and (c) 785nm.*

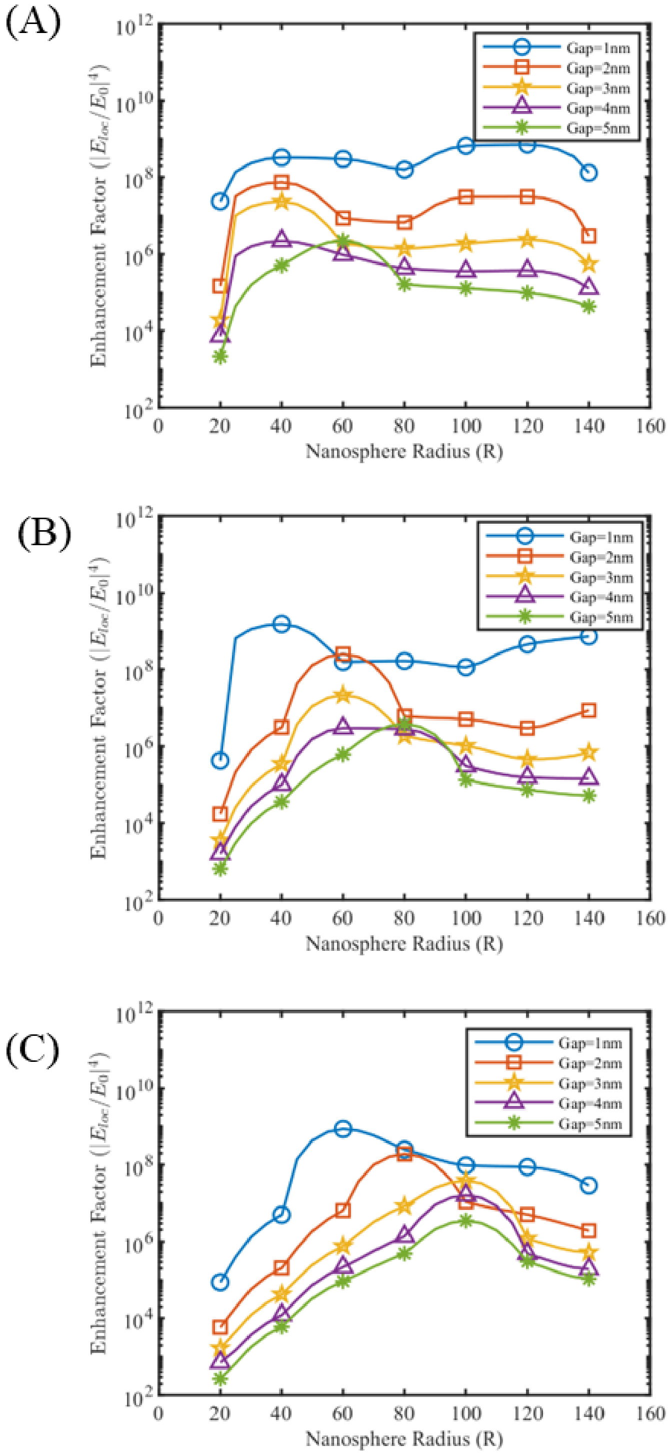

Figure S9: *Enhancement factor plots of silver nanotetramer orientation C at wavelength (a) 532nm (b) 633nm and (c) 785nm.*

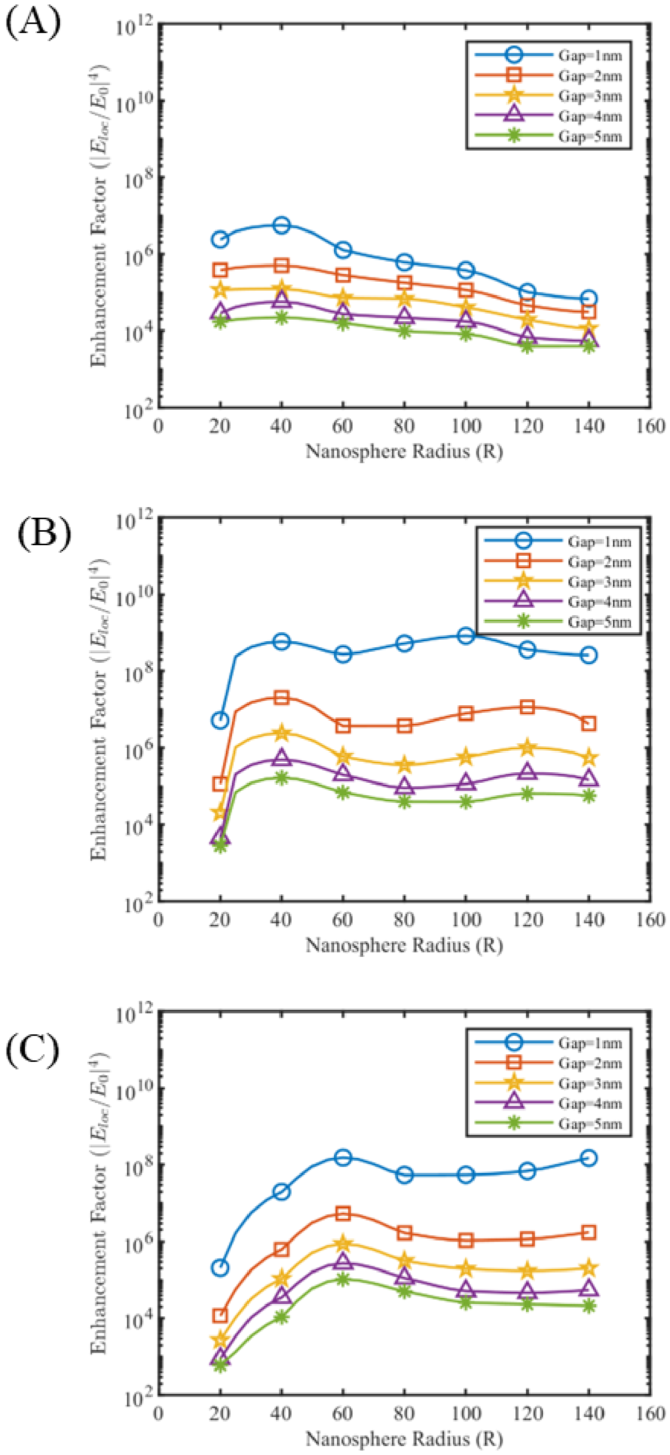

Figure S10: *Enhancement factor plots of gold nanotetramer orientation A at wavelength (a) 532nm (b) 633nm and (c) 785nm.*

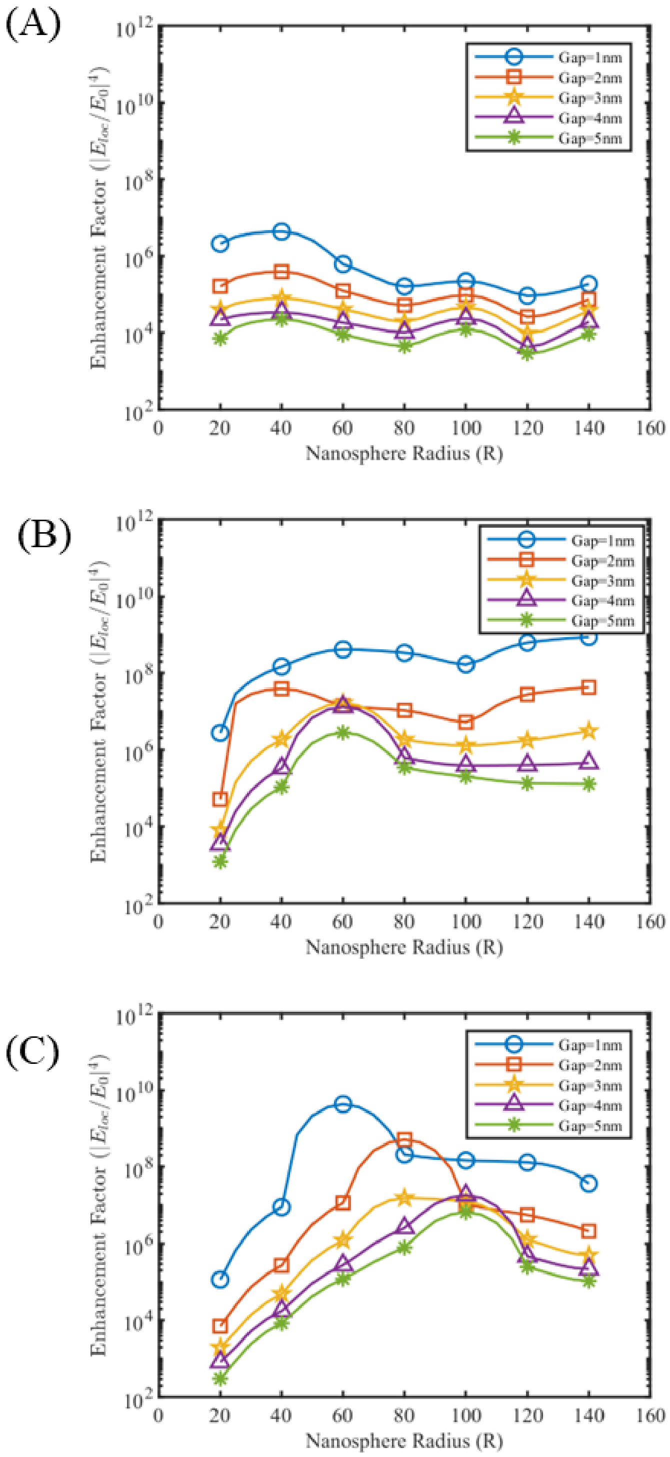

Figure S11: *Enhancement factor plots of gold nanotetramer orientation C at wavelength (a) 532nm (b) 633nm and (c) 785nm.*

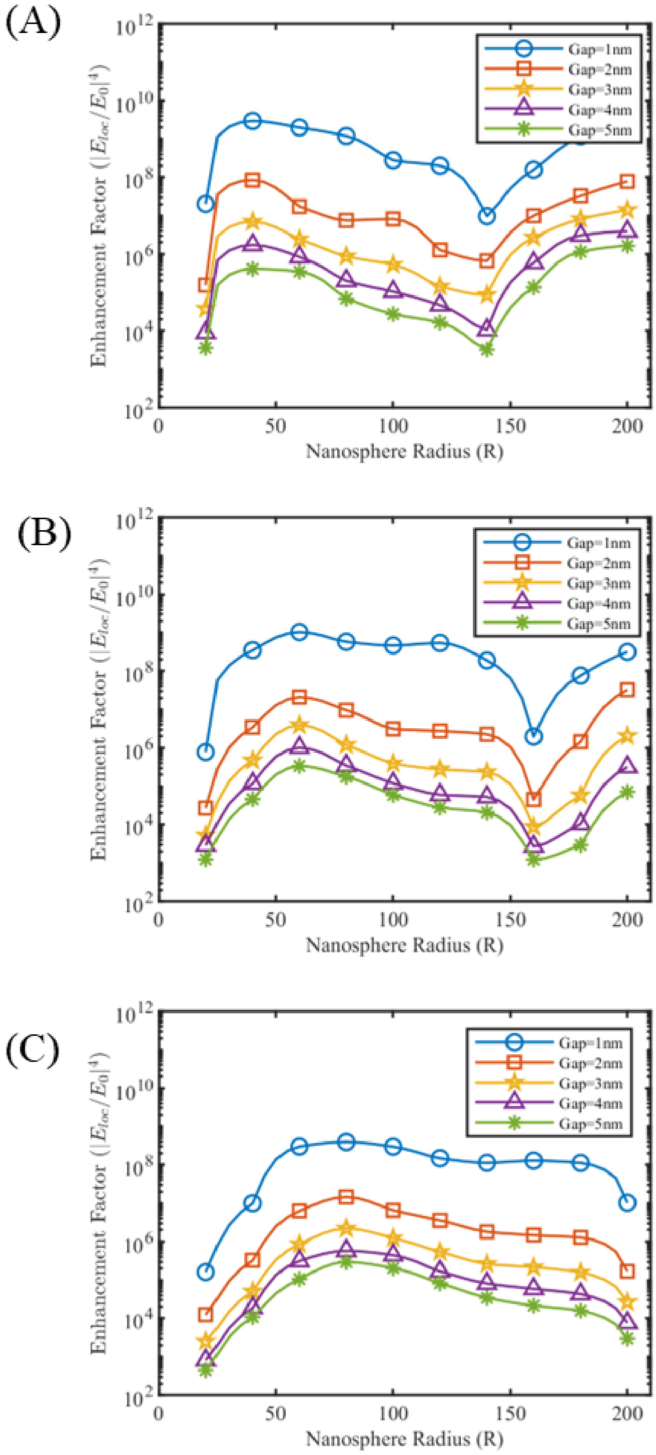

Figure S12: *Enhancement factor plots of hotspot between sphere 1 and 2 (middle hotspot) of silver nanotrimer orientation B at wavelength (a) 532nm (b) 633nm and (c) 785nm, demonstrating hotspot switching.*

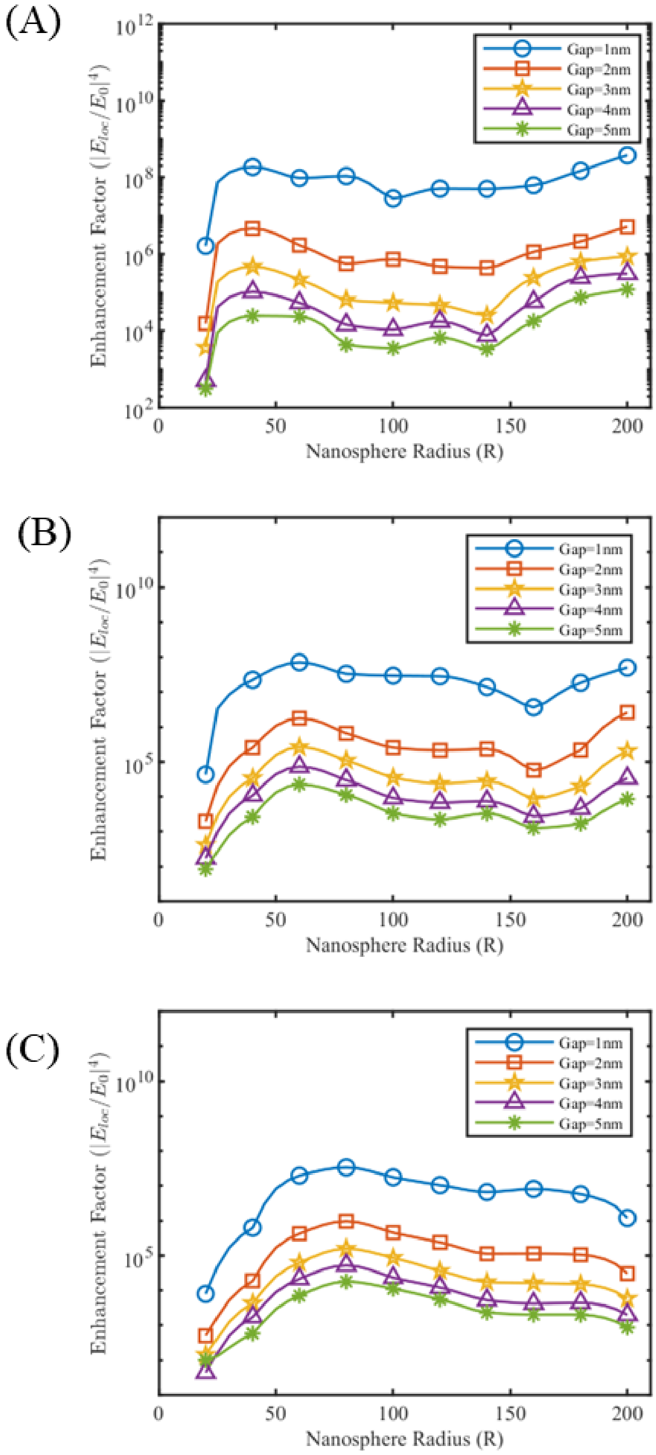

Figure S13: *Enhancement factor plots of hotspot between sphere 1 and 3 (side hotspot) of silver nanotrimer orientation B at wavelength (a) 532nm (b) 633nm and (c) 785nm, demonstrating hotspot switching.*

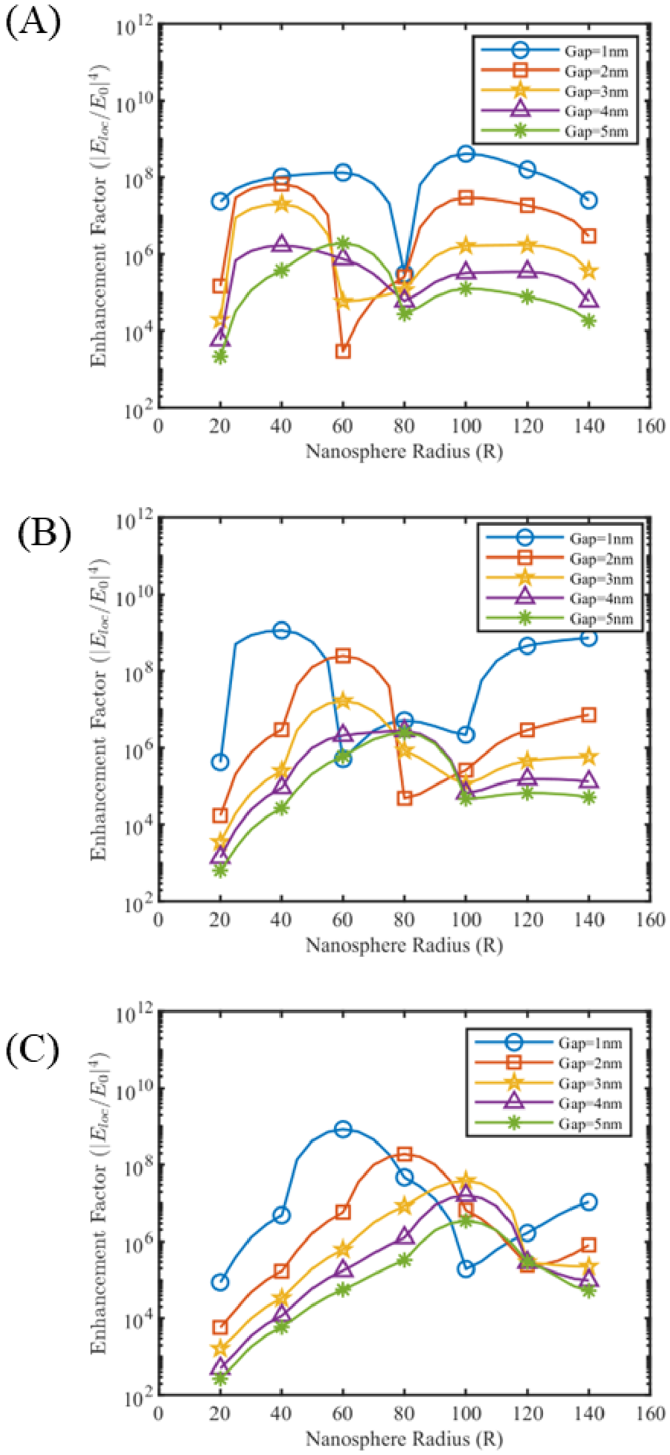

Figure S14: *Enhancement factor plots of hotspot 5 (middle hotspot) of silver nanotetramer orientation C at wavelength (a) 532nm (b) 633nm and (c) 785nm, demonstrating hotspot switching.*

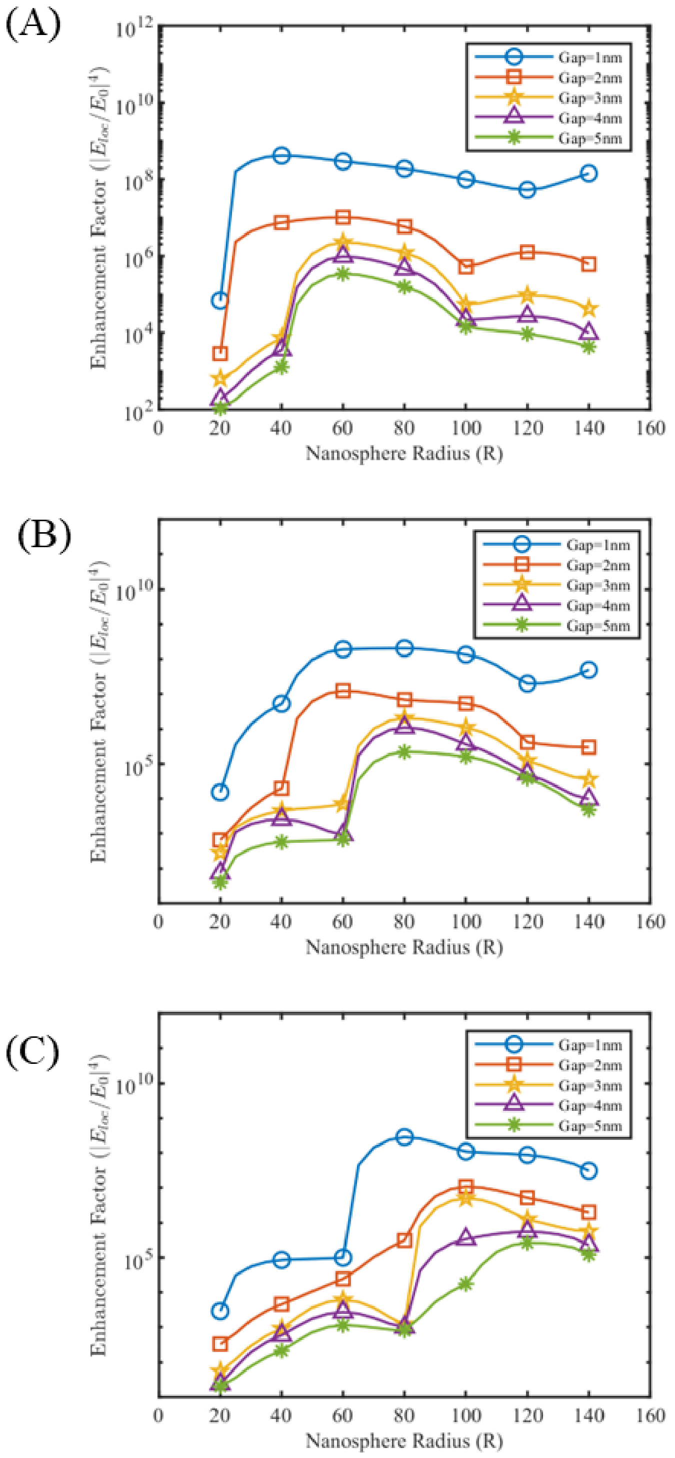

Figure S15: *Enhancement factor plots of hotspot 1 (side hotspot) of silver nanotetramer orientation C at wavelength (a) 532nm (b) 633nm and (c) 785nm, demonstrating hotspot switching.*

(A) (B)

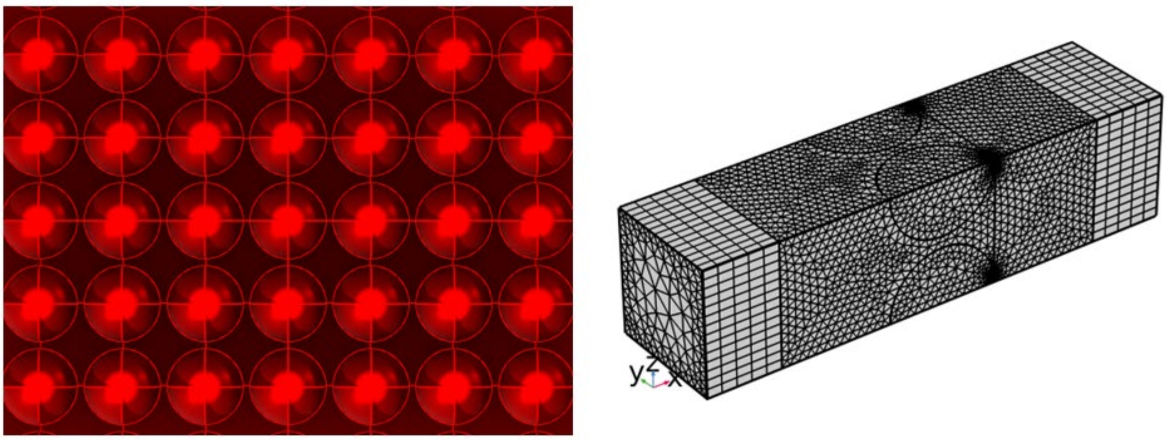

Figure S16: *(a) Top view of the model of periodic array of silver nanospheres over a silicon substrate. (b) meshing of the unit cell of periodic array of nanospheres.*

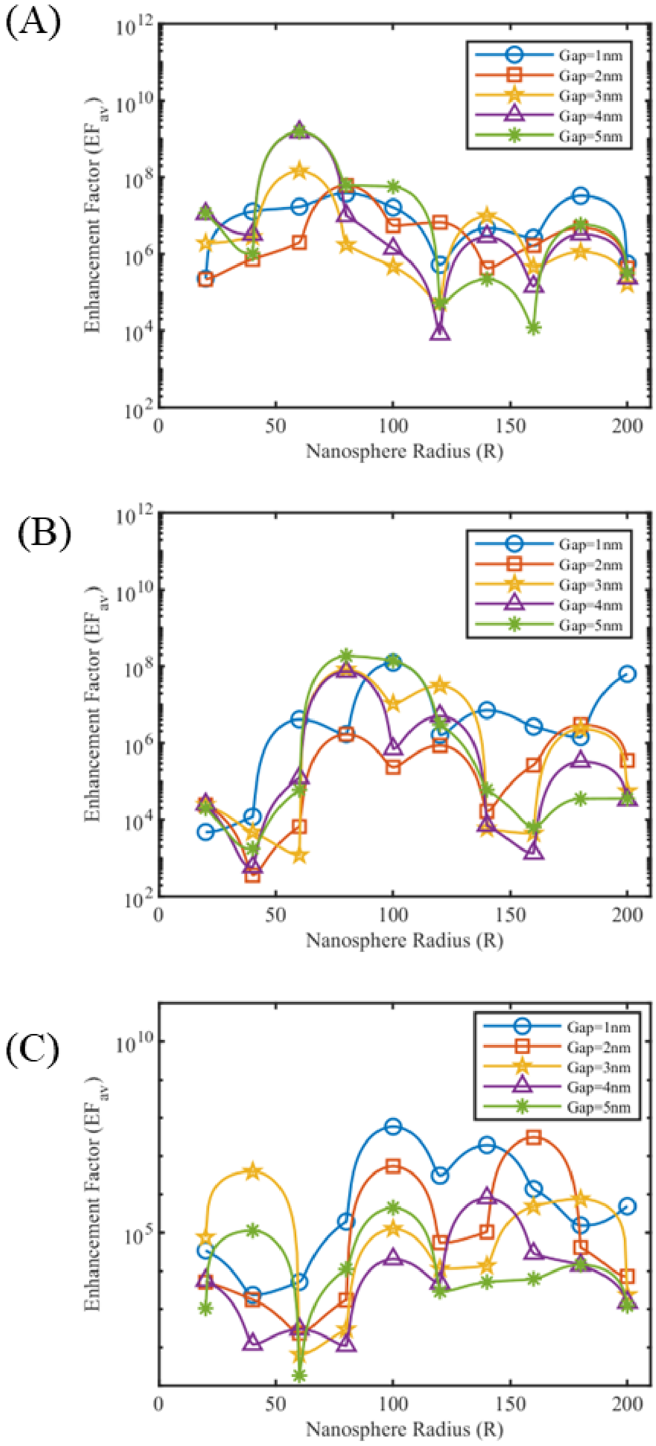

Figure S17: *Average enhancement factor plots of periodic array of silver nanospheres at wavelength (a) 532nm (b) 633nm and (c) 785nm.*

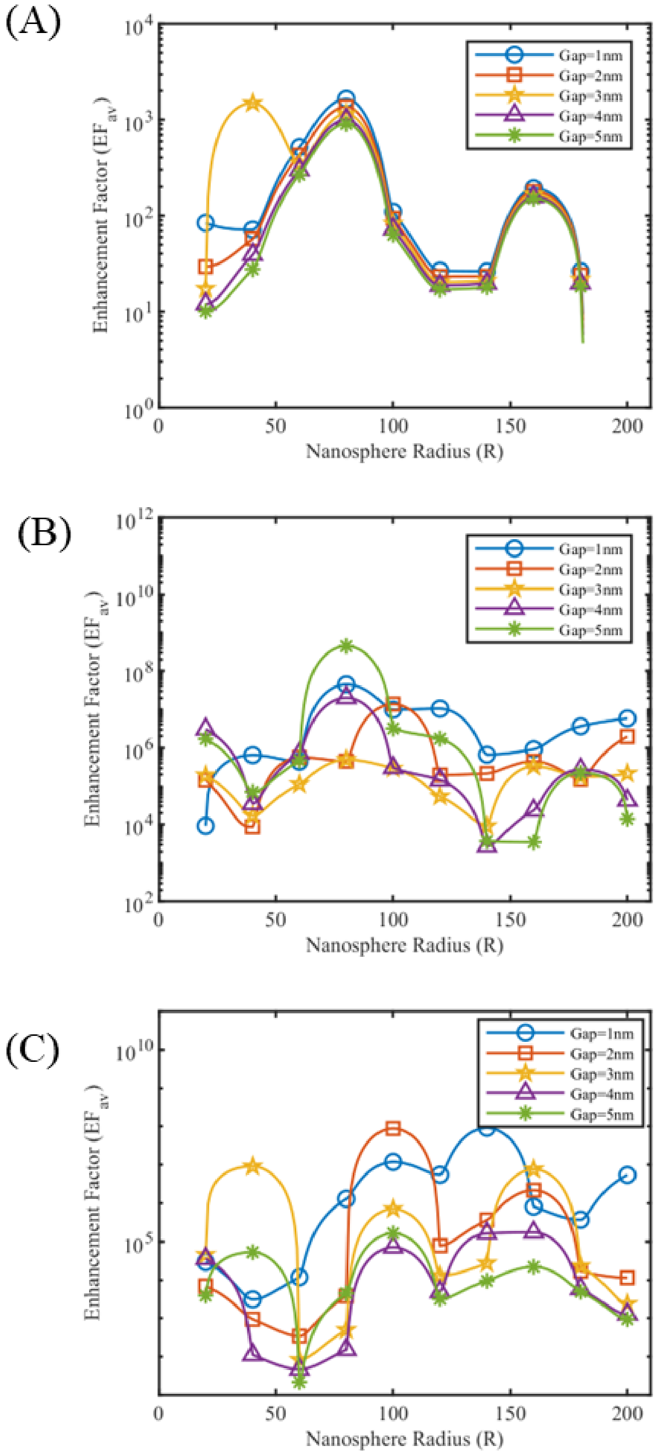

Figure S18: *Average enhancement factor plots of periodic array of gold nanospheres at wavelength (a) 532nm (b) 633nm and (c) 785nm.*
